# *Chlorella vulgaris* mutants with altered cell walls show increased permeability and enhanced extractability of intracellular molecules

**DOI:** 10.1186/s13068-025-02663-0

**Published:** 2025-06-05

**Authors:** Paolo Canteri, Claudia Battarra, Giulia Mandalà, Francesca Monti, Erika Bellini, Nora Hidasi, Zeno Guardini, Simone Ferrari, Roberto Bassi, Luca Dall’Osto

**Affiliations:** 1 Dipartimento di Biotecnologie, Università di Verona, Strada Le Grazie 15, 37134 Verona, Italy; 2 Dipartimento di Biologia e biotecnologie “Charles Darwin”, Sapienza Università di Roma, Piazzale Aldo Moro 5, 00185 Rome, Italy; 3 Accademia Nazionale dei Lincei, Palazzo Corsini, Via della Lungara 10, 00165 Rome, Italy; 4 Anton Dorhn Experimental Marine Station, Villa Comunale, 80121 Naples, Italy; 5 Present Address: Dipartimento Di Biologia, Università di Pisa, Via Luca Ghini, 13-56126 Pisa, Italy; 6 Dipartimento di Informatica, Università Verona, Strada Le Grazie 15, 37134 Verona, Italy

**Keywords:** Microalgae, *Chlorella vulgaris*, Cell wall, FACS/high-throughput fluorescence-activated cell sorting, Biomass extractability, Cell wall-weakened mutant

## Abstract

**Background:**

Large-scale cultivation of microalgae provides a carbon–neutral source of biomass for extracting valuable compounds and producing renewable fuels. Owing to their high metabolic activity and rapid reproduction rates, *Chlorella* species are highly productive when grown in photobioreactors. However, wild-type strains have some biological limitations that make algal bioproducts more expensive than those from more traditional sources. Domestication is thus required for improving strains. Engineering *Chlorella* species has been made difficult by their chemically complex and highly resistant cell wall, making transformation difficult. Cell wall also restricts diffusion of organic solvents; thus, limiting the extraction of valuable intracellular compounds. Obtaining strains with weakened cell wall is crucial to enhance the extractability of intracellular molecules, reducing the costs of biomass disruption, and to improve genetic transformation efficiency.

**Results:**

We developed a mutagenesis pipeline combined with single-cell fluorescence scanning on the microalga *Chlorella vulgaris* to identify mutants with altered cell wall properties. We used the fluorescent dyes erythrosin B and calcofluor white, as markers for cell wall permeability and for binding the structural polysaccharides of the cell wall, respectively. Flow cytometry with fluorescence-activated cell sorting was employed to enrich mutagenized populations with altered emission profiles. After a first round of mutagenesis, we found six mutants with significantly higher cell permeability to erythrosin B than the wild type (CWP lines) and altered cell wall structure and composition. A second round of mutagenesis on a selected CWP strain, followed by selection for lower calcofluor white signal, resulted in the isolation of CFW lines, which exhibited reduced mechanical resistance when the biomass was subjected to cell disruption procedures. This two-steps procedure allowed us to identify new mutant strains with both an increased cell wall permeability and a reduced mechanical resistance, making a novel step towards *Chlorella* domestication.

**Conclusions:**

This study demonstrated the feasibility of using mutagenesis and phenotypic selection based on flow cytometry screening to alter the cell wall of *C. vulgaris* and identify promising strains with improved traits for industrial applications.

**Supplementary Information:**

The online version contains supplementary material available at 10.1186/s13068-025-02663-0.

## Background

The large-scale cultivation of microalgae in photobioreactors (PBRs) has attracted significant interest over recent decades. This is because commercial algae production has been recognized as a renewable and environmentally sustainable strategy for generating feedstock [1]. Both microalgae and terrestrial plants drive photosynthetic reactions [2]; however, the simpler structure of microalgae allows for more efficient light-to-biomass conversion compared to plants [1, 3–5]. Consequently, cultivating microalgae represents a promising source of biomass for several industrial applications, including the production of bioactive compounds, recombinant proteins, livestock feed, biofuels, organic fertilizers, and biostimulants [6, 7]. Furthermore, the use of microalgae as cell factories can reduce production costs thus providing significant environmental benefits [8]; for example, by coupling microalgal biorefineries with CO_2_ abatement technologies or wastewater bioremediation, it is possible to recover nitrogen and phosphorus from industrial, municipal, and agricultural wastes [9–11].

Currently, algal biorefinery processes encounter limitations that hinder cost-effectiveness. These challenges encompass the expenses associated with the construction and management of PBRs, water pumping and mixing, axenic practices to prevent contamination of monocultures, biomass harvesting, cell disruption, and biochemical extraction. Notably, the latter significantly contributes to overall production costs [12]. Furthermore, extracting target products often results in a substantial amount of unused biomass, primarily composed of algal cell wall (CW) material. Valorizing this waste fraction is crucial for enhancing both economic and environmental sustainability [13].

To date, the most extensively studied microalga is the model organism *Chlamydomonas reinhardtii*, which has provided substantial insights into the regulation of metabolic pathways, enabling flexibility in response to external factors [14, 15]. Molecular biology toolkits and efficient transformation systems have been developed for this species [16, 17]; however, it holds little commercial interest [6]. On the other hand, other algal species are more suitable for industrial applications. *Chlorella vulgaris* (*Cv*), a freshwater unicellular Chlorophyta, has emerged as one of the most widely cultivated microalgae globally due to its high resilience and productivity [18]. *Cv* finds applications in food, feed, antioxidant and polyunsaturated fatty acid production [19], and wastewater treatment [20]. A major hurdle in processing *Cv* is the extraction of desirable compounds [21]. The robust CW of *Chlorella* species form a significant barrier to extraction [22], often necessitating harsh chemicals or high energy inputs [23]. Moreover, although the complete *Cv* genome has recently been sequenced [24], its rigid CW hampers the development of reproducible transformation protocols [25], posing major challenges to its engineering, which is crucial for the economic viability in the microalgal industry.

Recalcitrance is influenced by the composition and structure of the CW, as well as the biomass processing methods. The previous studies have explored physico-chemical or enzymatic treatments to reduce CW recalcitrance and enhance biomass extractability [26, 27]. These treatments included the use of organic solvents [28], strong acids [29], hydrogen peroxide oxidation [30], ultrasonication [31], microwaves [32], pulse electric fields [33] and hydrolytic enzymes [34]. However, often, these methods are not cost-effective and require intensive energy input [22, 33]. Generally, milder methods are preferred aiming to preserve the functionality of algal biochemicals [21]. Nonetheless, the structure and composition of the CW in *Cv* are highly dynamic across different growth stages, and variations in environmental conditions can further impact extraction efficiency [35].

Thus, identifying CW-deficient mutants is essential for reducing CW breaking costs and promoting genetic transformation efficiency in *Cv*. In other species, mutagenesis and phenotypic selection have been utilized to isolate microalgae with altered CW composition. Mutant strains of *Chlamydomonas reinhardtii*, exhibiting defects in CW biogenesis, were identified by scanning plates under a dissecting microscope [36]. One notable strain is CW15 [37], which has been found to be easier to transform than the wild type. In addition, *Haematococcus pluvialis* CW-deficient strains, isolated using FITC-conjugated lectins to profile changes in CW sugar compositions, demonstrated improved extraction efficiency [38]. Mutants targeting the cellulose synthase gene were generated using the CRISPR/Cas9 approach in *Nannochloropsis salina*. These mutants displayed a thinner CW and greater susceptibility to mechanical stress, which resulted in enhanced lipid extractability [39].

No mutants affecting the CW composition have yet been reported in *Cv* to date. While *Cv* is one of the most studied species, the structure and biogenesis of its CW are still not fully understood. [40] identified three distinct CW structures (Type I, II and III) and, after the latest taxonomic revisions, *Cv* has been reassigned to a Type III CW structure—namely a microfibrillar single layer. Transmission electron microscopy (TEM) has shown that the *Cv* CW consists of a single microfibrillar layer during the early growth phase; however, cells rapidly develop a three-layered structure characterized by a thick outermost layer (also referred to as hair-like fibers) surrounding a fine inner layer, which are separated by an electron-translucent interspace [41, 42]. It has been reported that the *Cv* CW is composed of 20–25% neutral sugars, 15–20% uronic acids, 7–17% glucosamine, and 6–10% protein [43]. However, reports on composition vary significantly, which may be attributed to differences in taxonomic classification, cultivation conditions, or cell states [41]. Enzymatic treatments have demonstrated that *Cv* cells are sensitive to chitinases and lysozymes, both of which degrade polysaccharides containing glucosamine [44]. A stepwise extraction of the *Cv* CW under increasingly strong conditions revealed that each fraction released different ratios of proteins and carbohydrates, helping to identify factors that contribute to the robustness of the CW [45]. Notably, the presence of chitin or chitin-like polymers was inferred from the glucosamine detected in strong alkali extracts, while the detection of glucose in strong acidic extracts indicated the presence of highly ordered starch or cellulose.

Domesticating microalgae for industrial biorefineries requires the introduction of traits that weaken the tough CW to (i) enhance the extraction yield of desirable compounds, and (ii) promote genetic transformation efficiency. However, it is crucial to evaluate whether manipulating CW strength negatively impacts biomass yield in a PBR environment, once weighed against the reduced costs of biomass processing. In this study, we report on the development of CW-deficient strains of *Cv* through two rounds of mutagenesis followed by fluorescence-activated cell sorting (FACS) selection. The first selection round yielded 6 mutant lines that exhibited increased permeability to the fluorescent dye erythrosin B and alterations in average CW thickness and/or composition, while their CW robustness remained unaffected. The second round of mutagenesis and selection led to the identification of strains with improved protein extraction efficiency. Importantly, the selected strains did not show impaired growth rates when cultured in a lab-scale PBR. Overall, these results demonstrate the feasibility of screening for CW mutants in *Cv* and resulted in domesticated algal strains that could positively influence the cost of downstream processes, such as cell disruption.

## Methods

*Strains and culture conditions.* The WT strain of *Chlorella vulgaris* (*Cv*) was acquired from the SAG Culture Collection of Algae (Goettingen University, Germany) as strain number 211-11p. Cells were maintained on TAP agar plates [46] and cultured in either minimal (BG-11) [47] or nutrient-rich (TAP) media, in shaking flasks (120 rpm) receiving top illumination of 80 μmol photons m^−2^ s^−1^, under a 16/8 h light/dark cycle at 24 °C (control condition). For synchronizing growth, conditions were adjusted to 200 μmol photons m^−2^ s^−1^, with a 12/12 h light/dark and 28/18 °C cycles [48]. Irradiance was provided by warm-white LEDs (4000 K). Cultures were harvested during the logarithmic growth phase (about 1–3·10^7^ cells mL^−1^) for all the measurements. The osmotic stress resistance of selected mutants was evaluated by spotting serial dilutions of a concentrated culture onto TAP-agar plates containing 0 to 500 mM NaCl and maintained in the light for 14 days.

*Cell count*. Cell density was assessed using an improved Neubauer hemocytometer and the Countess II FL cell counter (Life Technologies).

*Thermal treatment and enzymatic digestion of Cv cells.* Algal cells were washed twice, resuspended in phosphate-buffered saline pH 7.2 (PBS) upon centrifugation (3500 ×*g*, 3 min), then used to prepare samples with varying ratios of living (L) to dead cells: 100% L, 50% L, 0% L. Dead cells were obtained by heating cells at 100 °C for 15 min. For the enzymatic digestion of CW, *Cv* cell suspensions (1·10^7^ cells mL^−1^) were treated in a reaction buffer (0.1% w/v MES pH 5.5, 50 mM CaCl_2_, 0.6 M Sorbitol) with the following enzymes (from Merck): lysozyme 20.000 U mL^−1^ (62,971–106-F), chitinase 0.0025 U mL^−1^ (C6137-25), sulfatase 10 U mL^−1^ (S9626-10 KU), cellulase 9 U mL^−1^ (22,178-25G), according to [44]. The digestion was conducted at RT in the dark, for 16 h with gentle shaking.

*Staining of microalgal cells*. Fluorescent probes were employed to detect changes in the permeability of the *Cv* CW. After washing twice with PBS and harvesting by centrifugation, the algal cells were resuspended in PBS (5·10^6^ cells mL^−1^) and incubated for 10 min in the dark with specific fluorescent dyes. These included: Fluorescein diacetate (FDA, Merck F7378) 4 µg mL^−1^, Erythrosin B (EB, Merck 87,613) 2.5 µg mL^−1^, SYTOX green 50 µM (SG, Invitrogen S7020). Cells stained with EB and SG were washed 3 times with PBS to remove excess dyes. The staining with FDA was performed subsequently at the EB staining. Fluorescence emission was measured at RT on cells resuspended in PBS using a multiplate fluorimeter (Infinite Tecan 200 PRO): FDA, λ_exc_ 488 nm, λ_emis_ 519 nm; EB, λ_exc_ 526 nm, λ_emis_ 560 nm; SG, λ_exc_ 488 nm, λ_emis_ 529 nm. The staining with calcofluor white (Cf, Merck 18,909) was performed upon mixing 1% w/v Cf solutions with 5·10^6^ cells mL^−1^ washed cells, fluorescence analysis was performed using Leica DM4 DFC7000 T fluorescence microscope (Leica Biosystems, Germany). Leica Application Suite (LAS, X version 3.1.1.15751) software and Leica DFC7000 T camera (Leica Biosystems) were used for image acquisition with a 40 × objective. λ_exc_ 330–385 nm, λ_emis_ 430 nm. The fluorescence analysis was performed using ImageJ software.

**Fig. 1:**
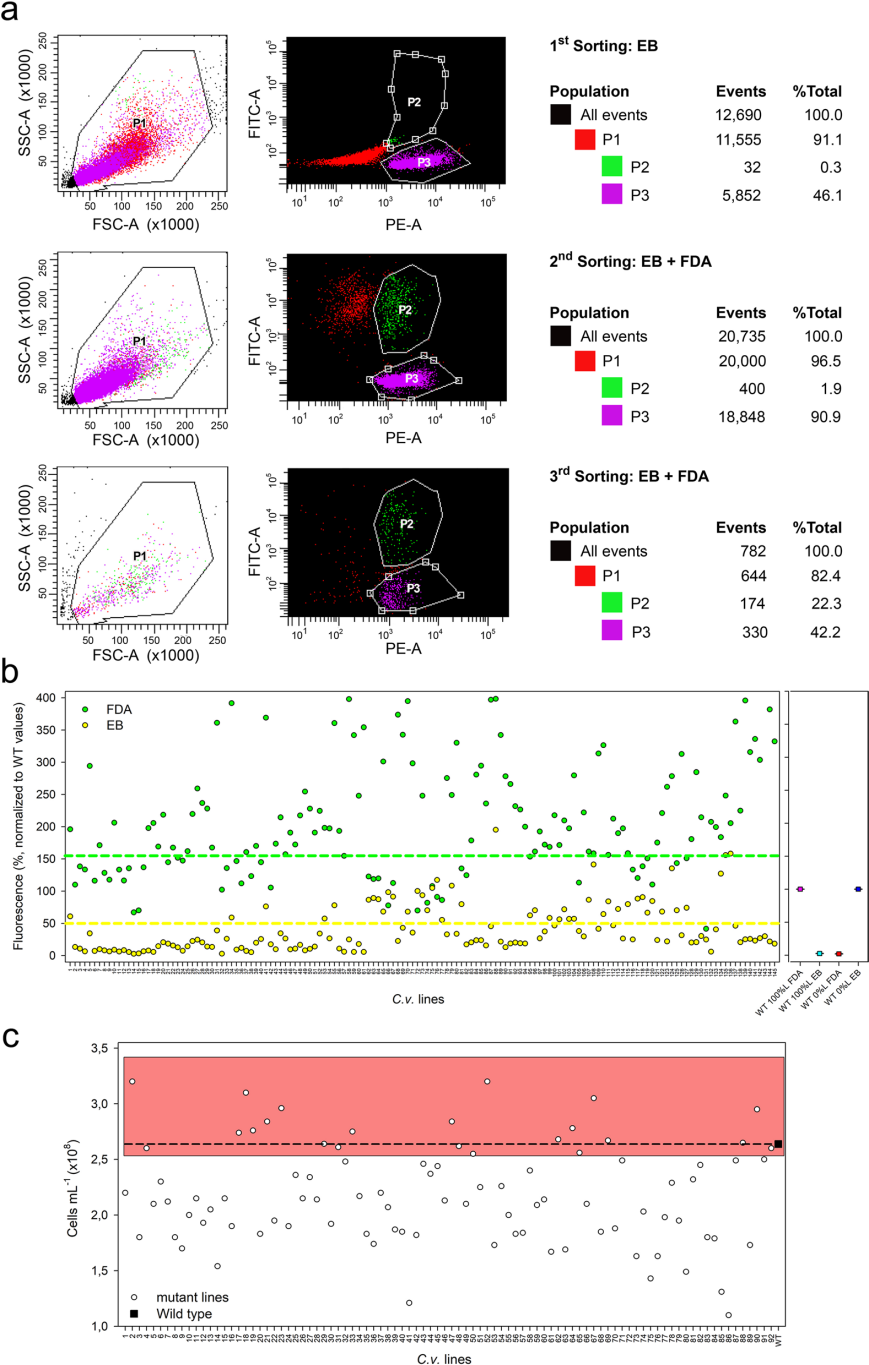
Screening strategy for isolating *C. vulgaris* mutants with increased cell wall permeability (CWP). **a** Flow cytometry generated the dot plots of algal cells from a population mutagenized with EMS. Cells were stained with selective probes, and high-fluorescent cells were sorted out by flow cytometry: (*upper panel*) first sorting, focused on Erythrosin B (EB) emission, using the phycoerythrin (PE) channel for EB detection (λ_exc_ 526 nm, λ_emis_ 560 nm); (*middle panel*) second sorting, conducted on the previously sorted P2 population for both Fluorescein diacetate (FDA) and EB, within the EB-emission-selected population, with FITC channel capturing FDA fluorescence (λ_exc_ 488 nm, λ_emis_ 519 nm)*;* (*lower panel*) third sorting, based on both FDA and EB emission levels. Two dot plots were produced for each sorting: (*left graph*) distribution of granularity and size of cells detected via forward scatter (FSC) and side scatter (SSC); (*right graph*) distribution of cells based on the fluorescence detection via the PE- and Fluorescein isothiocyanate (FITC)-channels. Gating: P1 for total selected cells, P3 for dead cells with high EB and low FDA signal, and P2 for cells with permeable CW, with high EB and FDA signals. **b** 145 single colonies were cultured in liquid minimal BG-11 medium. Samples (5·10^6^ cells) were stained with FDA and EB, and the fluorescence yield of each was measured. Inner controls included 100%L (WT living cells only) and 0%L (WT dead cells only), treated with EB and FDA. Controls included fully viable (100%L) and dead (0%L) WT cells. Strains with FDA emission at least 50% higher than WT100%L (green line), and EB yield at least half that of WT0%L (yellow line), were further selected, resulting in 92 lines. **c** The WT and selected mutant lines were cultured on BG-11, cell density was measured after 6 days, and strains surpassing 2.5·10^8^ cells mL^−1^ (pink box) were selected. This process isolated 22 putative CWP mutants

*Mutagenesis and screening protocols.* Mutagenesis was conducted following previously established methodologies [49]. WT *Cv* cells were collected by centrifugation (3500 ×*g*, 3 min), resuspended in fresh BG-11 medium to 5·10^7^ cells mL^−1^, and treated with increasing concentrations (0%–1.6%–1.8%–2.0%–2.2%–2.4%–2.7%–3%–3.4% *w*/*v*) of ethyl methanesulfonate (EMS). Following 2 h in the dark, cells were washed twice with 10% (w/v) sodium thiosulfate to inhibit light-activated DNA repair. A survival curve helped establish the mutagen concentration required to achieve approximately 5–10% cell viability. Cells were then plated with a 100-fold dilution on minimal BG-11 solid medium and exposed to 80 μmol photons m^−2^ s^−1^, with single colonies appearing after 14 days. Selected lines were transferred to fresh minimal medium, then tested for EB permeability (CWP lines, Fig. 1b) or by cell disruption (CFW lines, Figure S10).

*Fluorescence-activated cell sorting (FACS)*. After mutagenesis recovery (RT, 24 h in BG-11 liquid medium), each population was harvested by centrifugation, resuspended in PBS (5·10^6^ cells mL^−1^) and subjected to FACS using a FACSAria Fusion flow cytometer (Becton Dickinson, USA). The cells treated with EB were subjected to two additional washing steps with PBS before the FACS selection. Excitation of Erythrosin B, FDA, Cf and chlorophyll utilized an argon laser at 526, 488, 395 and 561 nm, respectively, with emitted fluorescence detected at 560 ± 5 nm, 519 ± 5 nm, 430 ± 50 and 695 ± 40 nm. The forward scatter (FSC) and side scatter (SSC) signals were recorded simultaneously. A chlorophyll fluorescence intensity threshold was set to ensure that only viable cells were selected. Data analysis was performed with FlowJo software 7.6.1 (Tree Star, Inc. Ashland, OR, USA). Dot plots of FDA and EB fluorescence intensities from WT *Cv* cells determined gating for identifying CW mutants. Identified mutants appeared in the P2 gate of the mutant library and were sorted out by using the flow cytometer under “Purity” mode. These mutants were subsequently cultured on BG-11 agar plates supplemented with 50 μg mL^−1^ ampicillin, 2,5 μg mL^−1^ tetracycline and 0,3 μg mL^−1^ amphotericin B. In the second FACS and screening phase using Cf, mutants showing the lowest dye fluorescence were identified in the P6 gate.

*Transmission electron microscopy (TEM).* Cell suspensions (~ 1·10^5^ cells mL^−1^) were embedded in a gelatine hydrogel (5% w/v) and fixed in 3% glutaraldehyde in 0.1 M cacodylate buffer pH 6.9, for 24 h at 4 °C. Samples were negatively stained with 2% uranyl acetate on glow-discharged carbon-coated copper grids. TEM was carried out using a FEI Tecnai T12 electron microscope operating at 100 kV accelerating voltage. Images were captured using a Gatan 4000 SP 4 K slow-scan CCD camera at 80,000 × magnification. Image binning achieved a pixel size of 0.375 nm at the specimen level using GRACE software [50].

*FTIR spectroscopy*. Synchronized cultures grown in TAP medium were harvested during the exponential phase. After centrifugation (3000 ×*g*, 3 min), the medium was removed, and the cells were washed twice with sterile water. Each sample was counted using the Cell Counter (Countess II, Thermo Fisher Scientific, USA) and resuspended in sterile water to a final concentration of 1·10^7^ cells mL^−1^. For each sample, 20 µL spots of each cell suspension were deposited on an infrared-transparent BaF_2_ slide and dried overnight at RT under a laminar flow sterile hood. Mid-infrared absorption spectra were collected in transmission mode in the 1800–700 cm^−1^ range using a Vertex 70 Bruker Optics spectrometer coupled to a Hyperion 3000 vis/IR microscope equipped with a 15 × objective and a photoconductive MCT detector. For each sample, eight to ten point-by-point spectra were acquired at 4 cm^−1^ resolution over a 50 µm × 50 µm area by co-adding 64 scans (27 s acquisition time).

*CW extraction.* Approximately 30 mg of lyophilized biomass was frozen in liquid nitrogen and ground to a fine powder using a ball mill at 23.1 1/s for 1 min, with two 5.5 mm stainless steel balls per tube, repeated twice consecutively. To prepare the alcohol-insoluble residue (AIR), 750 µL of 70% aqueous ethanol was added to each sample, followed by mixing and centrifugation (10,000 ×*g*, 5 min). Subsequently, 750 µL of a 1:1 (v/v) chloroform/methanol solution was added to the pellet, which was mixed, centrifuged, and then resuspended in 250 µL of acetone, dried, and weighed.

Starch was removed using α-amylase (MP Biomedicals) at 12.6 U mg^−1^ dry biomass. Samples were resuspended in 750 µL of 0.1 M sodium acetate buffer at pH 5, heated for 20 min at 80 °C, then cooled on ice. Enzyme and 17.5 µL of 0.01% NaN_3_ solution were added, and the samples were incubated at 37 °C on a shaker for 24 h. They were subsequently heated to 100 °C for 10 min, centrifuged, the pellet was washed, resuspended in 250 µL of acetone, dried, and weighed.

To hydrolyze CW polysaccharides, 200 µL of 2 M trifluoroacetic acid (TFA) was added to the AIR, and hydrolysis was performed at 121 °C for 90 min. The hydrolysates were neutralized with 200 µL of isopropanol and dried under a nitrogen flow. This neutralization process was repeated three times, with isopropanol added each time before evaporating the alcohol-TFA mixture. The dried samples were dissolved in 200 µL of water and filtered with a 0.22 µm Spin-X filter through centrifuging (14,000 ×*g*, 5 min).

*Monosaccharide analysis.* The hydrolyzed CW samples underwent monosaccharide analysis by high-performance anion-exchange chromatography with pulsed amperometric detection (HPAEC-PAD), using an ICS-3000 chromatographer (Thermo Fisher Scientific, USA) equipped with a CarboPac PA20 column, 3 × 150 mm (Dionex, CA, USA). An isocratic elution with 6 mM NaOH at a flow rate of 0.34 mL min^−1^ for 22 min was used to separate neutral sugars and sugar amines, followed by a gradient up to 800 mM NaOH for 22 min to separate sugar acids. The column was equilibrated between samples for 13 min in 204.5 mM NaOH. A pulsed amperometric detector set to waveform A was used for detection, and monosaccharides were quantified using standard calibration curves.

*Cell disruption by ultrasonication.* This procedure was performed using a VC-750HV (20 kHz, probe 13 mm) ultrasonic processor, following the methodology outlined in [45]. Ultrasonication was performed on 0.5 g dry biomass in 25 mL of water, over a total duration of 30 min, with cycles of 5 s of ultrasonication followed by 15 s of rest to prevent overheating. Unbroken cells and debris were removed through centrifugation at 10,000 ×*g* for 10 min at RT.

*Cell disruption by glass beads*. According to the reference method [51], samples (1·10^8^ cells) were mixed with 300 µL of PBS and disrupted using glass beads in a bead beater (beads diameter 420–500 µm, BioSpec Products Inc., USA) at a speed of 5000 rpm for 20 s cycles, with 30 s intervals between cycles. Unbroken cells and debris were removed by centrifugation (10,000 ×*g*, 10 min at RT). A calibration curve was established to correlate the number of bead-beating cycles with the amount of protein extracted, for both WT and CWP1 cells (Figure S9).

*Colorimetric method for protein quantification.* Supernatants obtained from biomass treatments were analyzed for protein content, using the Bradford assay [52] or the BCA assay [53]. Total proteins were extracted from biomass treated with the enzymatic mixture E1 (see Figure S2) following 6 cycles of bead beating, and then extracted with a lysis buffer (6% SDS, 6 M Urea, 187 mM Tris–HCl pH 6.8).

*Growth analysis.* Growth experiments were conducted at 24 °C in a lab-scale PBR (MC 1000-OD, PSI, Czech Republic). The system was supplied with air supplemented with 2% CO_2_, and microalgae were exposed to 80 μmol photons m^−2^ s^−1^ continuous light.

*Statistics*. Statistical significance was assessed using one-way ANOVA, followed by either Tukey’s or Dunnett’s post hoc test. Error bars represent the standard deviation.

## Results

### Isolation of CWP lines, mutants of Chlorella vulgaris with altered cell wall permeability

To isolate *C. vulgaris* mutants with a CW altered as compared to the WT strain, we first developed an assay to screen a large population of mutagenized lines. We hypothesized that structural changes in the CW could alter its permeability properties. To evaluate this parameter, we utilized fluorescence from two probes: (i) Erythrosin B (EB), a polar dye that cannot penetrate the CW/membrane barrier and only enters dead cells, and (ii) SYTOX Green (SG), a DNA staining dye that typically cannot cross the CW. Additionally, we employed fluorescein diacetate (FDA), a probe that freely enters intact cells and is converted into fluorescein, which is retained in living cells due to its charged nature, while dead cells allow the probe to leak out. Therefore, these dyes can be used to stain *Cv* populations, enabling the assessment of both vitality and altered permeability through a fluorometric assay (Figure S1).

Samples consisting of varying ratios of living (L) and dead WT cells (100%L, 50%L, and 0%L) were treated with either SG or FDA, after which cell permeability and vitality were evaluated using a multiplate reader. This setting allows creating a reference (100%L as the maximum value for FDA and 0%L for EB or SG) and to verify the accuracy of the plate reader by simulating a mixed population of living and dead cells (50%L). As illustrated in Figure S2, SG, EB and FDA were effective in accurately distinguishing between living and dead *Cv* cells, with the proportional fluorescence correlating to the composition of the individual samples. The data enabled us to set fluorescence intensity thresholds: populations with intensity below the FDA threshold were considered as contaminated with dead cells, whereas samples exhibiting emissions above the EB and SG thresholds were deemed more permeable to the dyes with respect to the WT strain. We further investigated enzymatic hydrolysis of algal CW using the fluorescence assay. The results showed that degrading the CW with enzymatic cocktails increased staining relative to the 100%L sample, with the E1 mixture (lysozyme, chitinase, sulfatase) having the most significant effect on enhancing permeability to all probes (Figure S2). Consequently, cells displaying fluorescence intensity above the 100%L threshold for EB and SG were classified as permeable to the dyes.

The same populations of living and dead WT cells were utilized to establish and validate a flow cytometry-based cell sorting method. Living and dead cells can be distinguished from each other through FDA and EB staining, which indicates their markedly different CW permeability. These populations were employed to mimic CW mutants, aiding in the development of the FACS approach (Figure S3a, b). Side scatter (SSC) and forward scatter (FSC) amplitudes were used as indicators of cell size, allowing us to exclude potential false positives from aggregates or debris present in the culture. In the two-dimensional dot plot of FDA fluorescence intensity *vs.* SSC, living cells were distributed evenly in region P3, while the 0%L cells stained with EB fell in region P2. This differentiation indicates that groups of cells with discernible differences in staining can be separated effectively. Following this, a cell population treated with enzyme mix E1 (refer to Figure S2) was stained with both FDA and EB and subjected to flow cytometry analysis (Figure S3c). In the two-dimensional dot plot of FDA *vs.* EB fluorescence intensity, cells clustered in specific regions: within the selected population (P1), 20.8% of the cells were found in region P3 (blue dots), exhibiting high EB staining and low FDA retention, similar to dead cells. Conversely, region P2 (green dots) contained 14.9% of the population, representing cells that retained both probes, indicating increased permeability due to enzymatic digestion. The red dots represented cells with unaltered CW permeability, comprising 71.6% of the total population.

Based on the optimized staining probes and established FACS methods, we performed a screening of *Cv* CW mutants involving mutagenesis, staining, cell sorting, and phenotyping. Mutant libraries were generated through chemical mutagenesis with EMS at concentrations ranging from 1.6% to 3.4% (see Methods for details). The mutants were then recovered in fresh BG-11 medium and incubated in the dark for 24 h. Each library, containing approx. 3.8·10^7^ cells, were stained with both EB and FDA and subjected to FACS analysis.

In comparison to the WT cell population, where 96.1% of cells displayed low EB staining (Figure S4a, red dots), 4.1% of the mutant library generated with 2% EMS was found in the P2 region (Figure S4b).

To further refine the screening process, we implemented a two-step dye incubation procedure: first, the mutant libraries were stained with EB only and selected for high EB fluorescence (P3 region); this sub-population was then stained with FDA and sorted out twice through the P2 gate (Fig. 1a). A total of approx. 30,000 events—both positive to FDA and EB fluorescence—were sorted by gating at P2 and plated on BG-11 agar plates in low density. After 2 weeks, 861 colonies emerged (Table S1). A second screening was carried out by staining cell suspensions with both EB and FDA and establishing a minimum emission threshold for each dye. This ensured that most samples exhibited fluorescence intensity below the threshold (Fig. 1b). Cells were deemed permeable to the dyes if they met the following criteria: (i) FDA fluorescence was at least 50% higher than in the 100%L reference sample, and (ii) EB fluorescence was at least half that of the 0%L reference sample. Among the identified strains, 22 exhibited the highest growth rate under batch conditions (Fig. 1C), while 6 lines demonstrated increased permeability to the dye SG (Figure S5). These lines were selected and designated as CWP (cell-wall permeable) lines.

### Structural and spectroscopic characterization of CW in WT and mutant strains

**Fig. 2:**
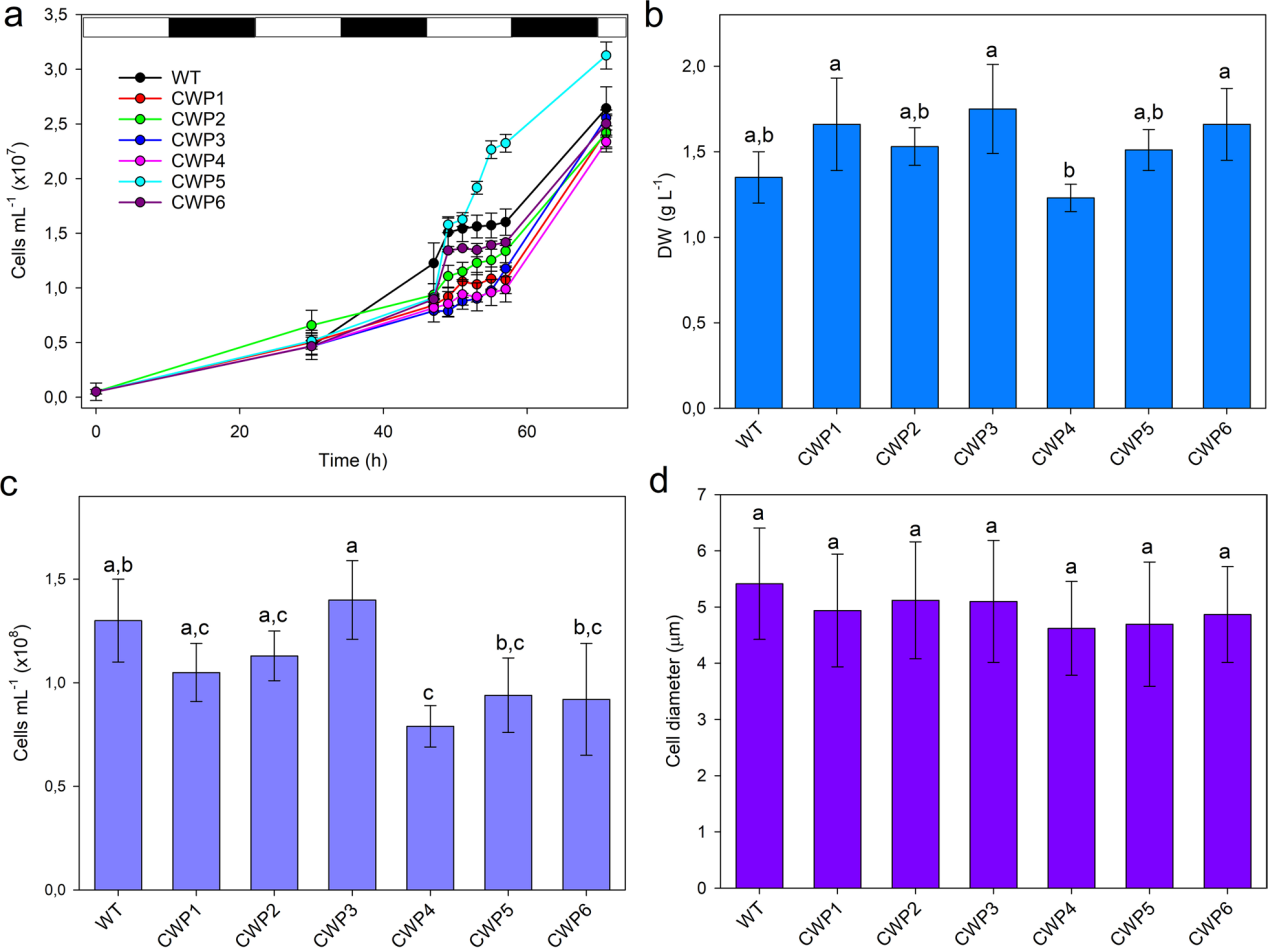
Characterization of CWP mutants in *C. vulgaris*. **a** Growth curves for synchronized *Cv* WT and CWP mutant lines. The cell density was measured using a cell counter for cultures grown at 28 °C during the light phase (12 h, 200 μmol photons m^−2^ s^−1^, indicated by white bars) and at 18 °C during the dark phase (12 h, indicated by black bars). Initial inoculum: 10^6^ cells mL^−1^, obtained after 2 weeks of preculturing under the same conditions. Data are expressed as mean ± SD, n = 5. **b**, **c** Dry biomass productivity and cell density value after 9 days of growth of mutants and parental strain in synchronized conditions. Error bars represent the deviation between replicates (*n* = 3) determined by ANOVA followed by Tukey’s post hoc (*p* < 0.05). **D** Average size of WT and mutant cells, measured after 74 h of growth in synchronized conditions. The sizes across all lines showed no significant differences (ANOVA followed by Tukey’s post hoc test at a significance level of *p* < 0.05)

CWP lines were thoroughly analyzed for growth rate and biomass productivity. This characterization of strains produced through chemical mutagenesis is essential to confirm that mutants do not suffer significantly reduced growth. Photoautotrophic growth was observed in a lab-scale PBR under a semi-batch cultivation system. As reported by [48], cell synchronization can be achieved under photoautotrophic growth by applying a temperature of 28 °C during the light phase and 18 °C during the dark phase, which leads to a doubling of cell numbers. Thus, batch cultures of *Cv* WT and CWP mutants were kept under these conditions (12 h light at 28 °C and 12 h darkness at 18 °C) during 2 weeks of preculturing to ensure a long-term adaptation. Subsequently, cells were used to inoculate a new culture, and cell numbers were measured using a cell counter (Fig. 2a). As expected, the WT strain maintained a relatively constant cell number during the third light phase, with a significant increase during the last dark phase, indicating synchronized cell division. Most mutant strains exhibited growth patterns similar to WT cells, where cell division paused and the cell number remained stable for several hours during the third day, before resuming and reaching comparable cell densities after 74 h of cultivation. In contrast, the CWP5 strain displayed a different growth pattern: cell division occurred even during the light phase, resulting in ~ 2.2 · 10^7^ cells mL^−1^ at the end of the third day, and reached about 3.1 · 10^7^ cells mL^−1^ by the fourth day, as compared to 2.6 · 10^7^ cells mL^−1^ in the WT. All genotypes exhibited dry biomass productivity after 9 days of growth (Fig. 2b), which roughly corresponded with their cell density values (Fig. 2c), and had cells of similar size when observed using bright-field microscopy (Fig. 2d).

**Fig. 3:**
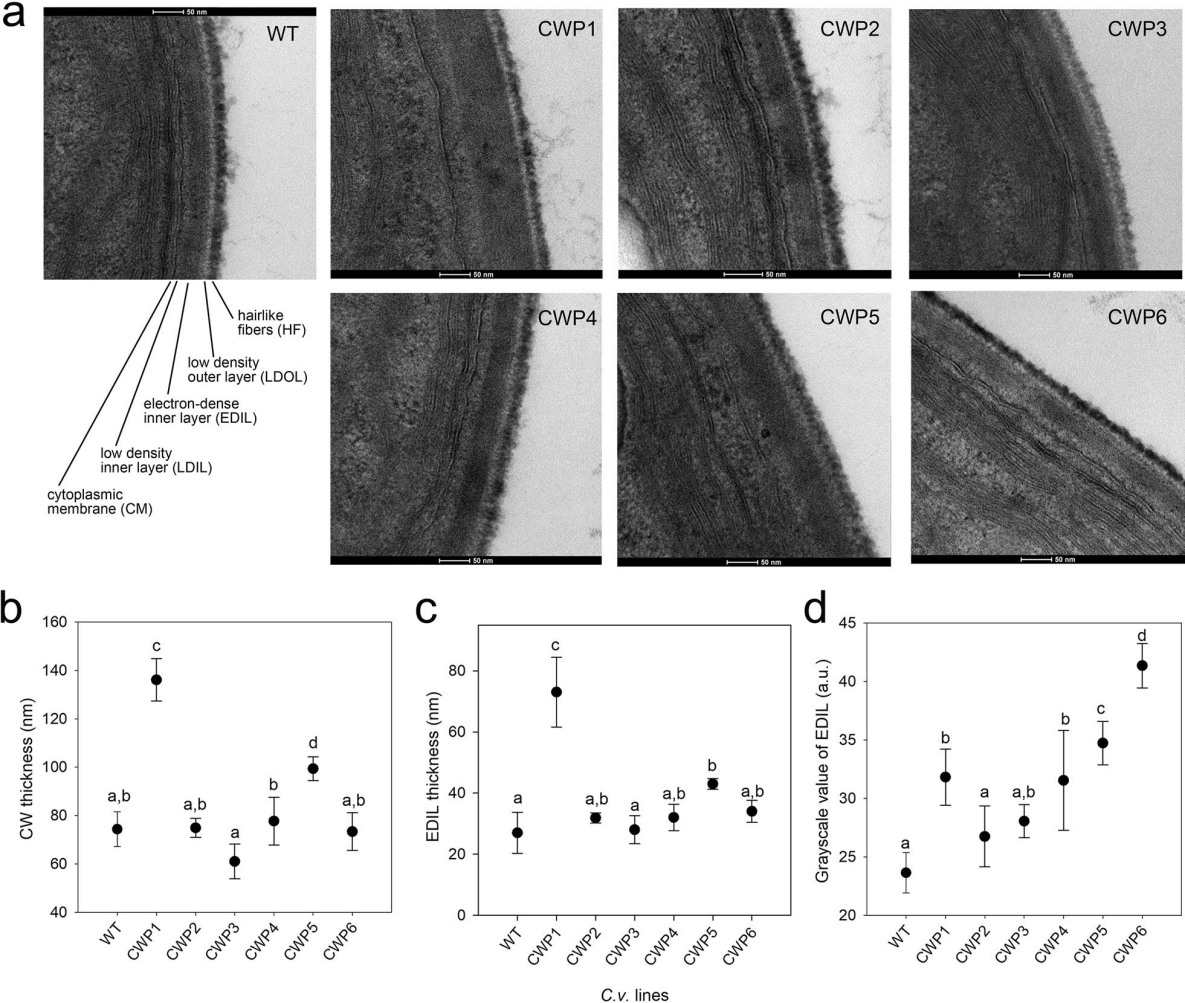
Transmission electron micrographs of WT and CWP cells. **a** Cells from synchronized cultures were collected after 6 h of light exposure, then fixed, embedded, and observed in thin sections at different magnification levels. The images presented are representative transmission electron micrographs of *Cv* WT and mutant cells. Notable features, including hairlike fibers (HF), low-density outer layer (LDOL), electron-dense inner layer (EDIL), and low-density inner layer (LDIL), are labeled. **B**–**D** Statistical evaluation of the morphological parameters of the cell walls from WT and CWP mutants*.* Graphs illustrate: **b** cell wall thickness, **c** thickness of the electron-dense inner layer, and **d** electron density of the EDIL portion, expressed as grayscale scale and normalized to the background. Data are shown as mean ± SD, *n* = 5. Values that are significantly different, identified through ANOVA followed by Tukey's post hoc test at a significance level of p < 0.05, are indicated by different letters

Using transmission electron microscopy (TEM), we examined how mutations affect the CW ultrastructure (Fig. 3). In the CW of WT cells, four distinct domains are visible: an outer low-density layer (LDOL) and an electron-dense inner layer (EDIL), which is separated from the cytoplasmic membrane by a thinner, less electron-dense layer (LDIL). Hairlike fibers (HF) extend from the outer layer (Fig. 3a). All mutant lines retained the four-layer organization seen in WT cells; however, some mutations altered the structure of these layers. Specifically, strains CWP1 and CWP5 showed a thickened CW (Fig. 3b), primarily due to swelling in the EDIL domain (Fig. 3c). The electron density of the CW was assessed by grayscale values, which indicate the degree of X-ray beam attenuation by the tissue: darker pixels (values close to 0) indicate structures with less beam attenuation, whereas lighter pixels count for higher attenuation. Notably, mutants CWP1, CWP4, CWP5, and especially CWP6 exhibited a decrease in the electron density of the EDIL layer (Fig. 3d), as shown by the grayscale values.

**Fig. 4:**
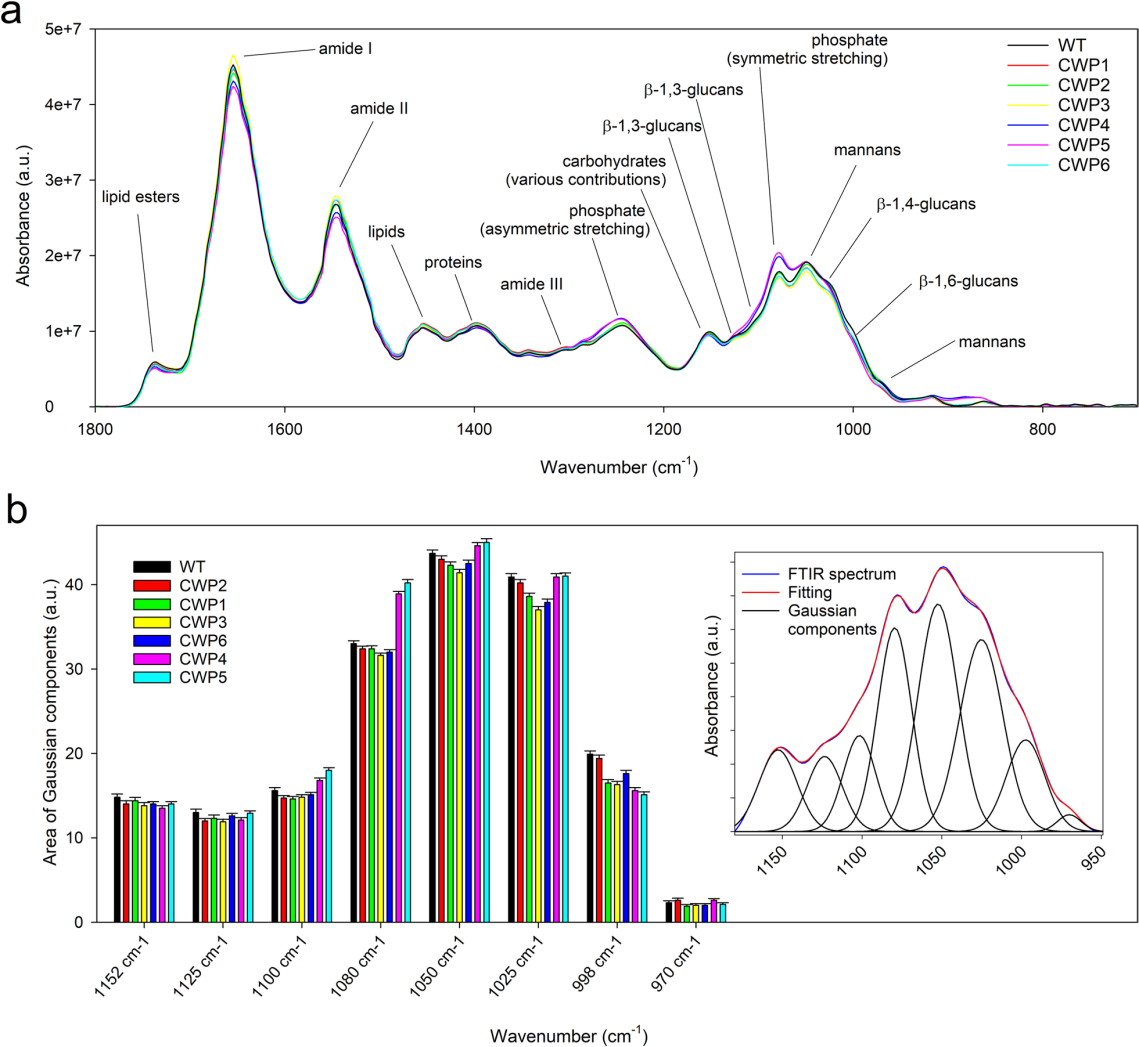
FTIR Spectroscopy of WT and CWP Cells. **a** Average baseline-corrected and area-normalized absorbance spectra for WT and CWP mutant lines in the 1800–700 cm^−1^ range. Main absorption bands are labeled for clarity as discussed in the text. **b** Areas of the eight Gaussian bands utilized in the curve fitting of the WT and CWP absorption spectra in the 1175–950 cm^–1^ range. Error bars on the intensities of the absorption bands were estimated from the RMS error of the fit by propagating the relative uncertainties in the heights and widths of the Gaussians. The inset shows the curve fitting (black lines for the individual Gaussians and a red line for their sum) as obtained for the CWP1 strain spectrum (blue line)

Fourier Transform Infrared Spectroscopy (FTIR) in the mid-infrared range is an accurate and nondestructive technique that provides an overall evaluation of the cell and CW composition with minimal sample preparation. It includes information on functional groups or bonds in biochemical components (proteins, lipids, nucleic acids, and carbohydrates) and their relative contributions [54 and references therein]. The spectra collected on the WT cells and the six mutants were baseline corrected and, for each cell type, averaged and area normalized using the Bruker OPUS 7.5 software (Fig. 4a). Area normalization ensures that the obtained information is related to the overall biochemical composition, i.e., normalized to the same amount of biological material.

Most meaningful absorption bands related to the CW components [54] overlap in the 1180–950 cm^−1^ wavelength range. Their positions can be determined from the minima in the second derivatives of the average spectra. Eight absorption bands were identified. A significant result of our study is that, based on previous FTIR studies on cell and CW components of various yeast strains [54], we were able to assign each absorption band to its corresponding main CW component, as shown in Table S6b. All the absorption bands in this region are related to major parietal polysaccharides: mannans (mainly around 1045/1050 cm^−1^ and 968 cm^−1^) and β-glucans (β−1,3 mainly around 1130 cm^−1^ and 1105 cm^−1^; β−1,4 around 1025/1030 cm^−1^; and β−1,6 around 995/998 cm^−1^). Another band at around 1150 cm^−1^ can be attributed to carbohydrates, including chitin components. Conversely, the band at around 1080 cm^−1^, related to the phosphate group (symmetric stretching), although also representing phospho-mannans present in the outer part of the CW, can be predominantly associated with nucleic acids in the cytoplasm and phospholipids of the membrane [54].

Visual inspection of the spectra (Fig. 4a) indicates that the average spectrum of CPW2 is practically identical to that of the WT, while CPW4 and CPW5 exhibit significant differences, primarily due to the 1080 cm^−1^ phosphate band, which is mainly associated with the cell cytoplasm and membrane.

To gain further insight into the subtle differences among the absorption spectra of the various strains, a more detailed investigation of the relative contributions of each biochemical component was conducted using curve fitting with the Bruker OPUS 7.5 software. Fits were performed coherently on all spectra in the 1180–950 cm^−1^ range, after baseline correction and area normalization in this restricted spectral region. The least-squares minimization method was employed with eight Gaussian bands, whose starting positions were identified from the minima of the second derivatives of the spectra. The intensities of each absorption band in the WT cells and the six mutant strains are displayed as histograms in Fig. 4b. The inset shows the curve fitting obtained for the CPW1 strain. Error bars on the intensities of the absorption bands were estimated from the RMS error of the fit by propagating the relative uncertainties in the heights and widths of the Gaussians.

As expected, the main difference among the spectra is attributed to the 1080 cm^−1^ phosphate band. Absorption bands more directly related to the biochemical composition of the CW can be visually emphasized by comparing the sum of all fitted Gaussian bands except for the 1080 cm^−1^ band for each strain (Figure S6A). By examining Figs. 4b and S6A, the main differences detected by FTIR are evident in the 998 cm^−1^ absorption band, which is slightly lower in CWP1, CWP3 and CWP6 as compared to WT and CWP2, and much lower in CWP4 and CWP5. In addition, the 1025 cm^−1^ band is also slightly lower in CWP1, CWP3 and CWP6, while the 1105 cm^−1^ and 1050 a cm^−1^ bands are slightly higher in CPW4 and CPW5.

### Characterization of CW composition and mechanical resistance in WT and mutant strains

**Fig. 5:**
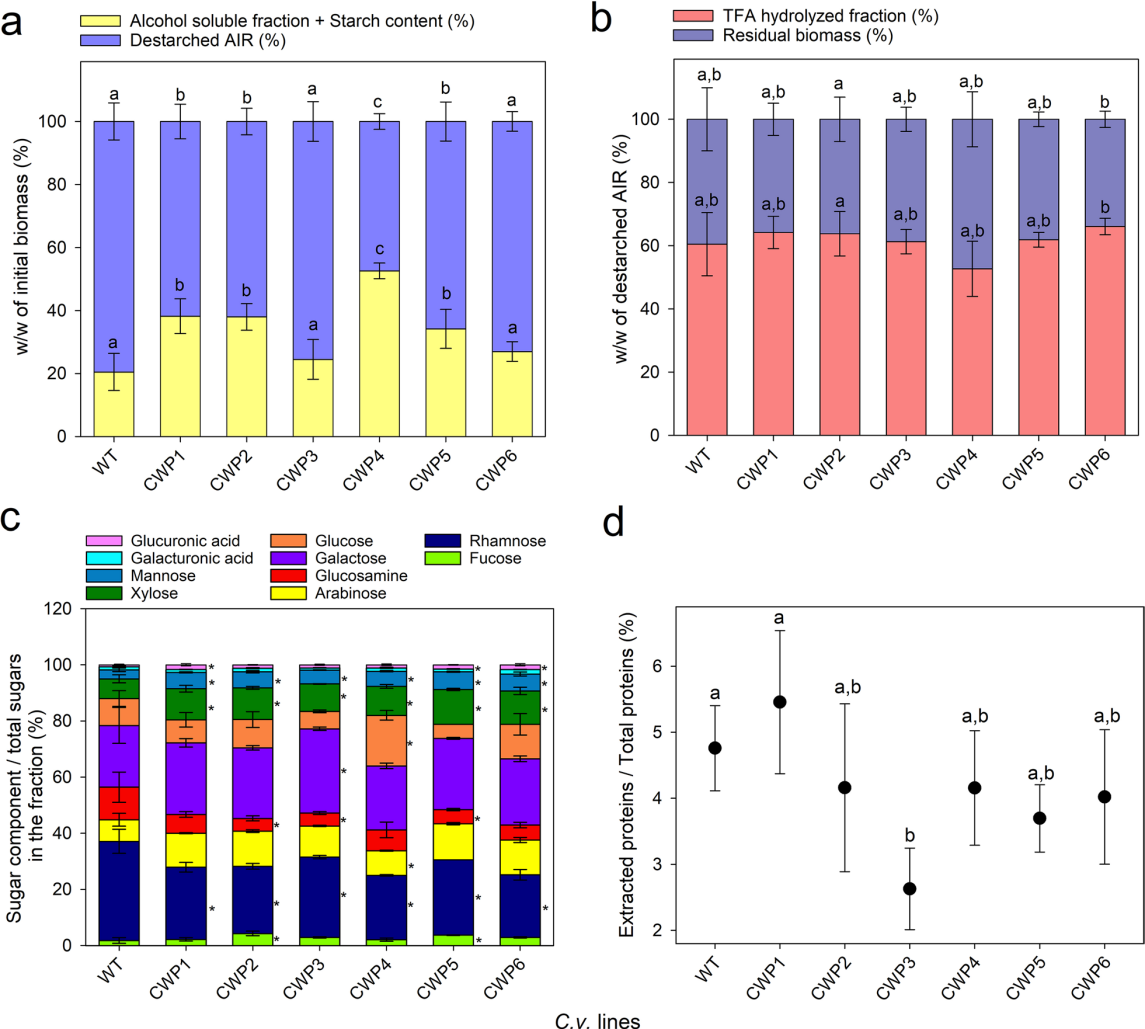
Characterization of cell wall composition and strength of CWP lines. **a**, **b** Percentage weight of the various fractions compared to the initial biomass weight (**a**) and to the alcohol-insoluble residue (AIR) weight (**b**). The bars represent the mean ± SD (*n* = 3). Different letters indicate statistically significant differences within each fraction at *p* < 0.05, as determined by one-way ANOVA followed by Tukey’s post hoc test. **c** Percentage weight of the sugar component relative to the total sugars weight in the fraction. The bars indicate the mean ± SD (*n* = 3). Asterisks indicate statistically significant differences compared to the WT, according to one-way ANOVA followed by Dunnett’s post hoc test. **d** Protein extraction efficiency from algal biomass. To assess the mechanical resistance of cell walls, wet biomass (1·10^8^ cells) was disrupted using a bead beater with glass beads. The amount of protein in the supernatant after disruption is shown relative to the total protein content in the biomass. Protein levels were quantified using the Bradford assay. Different letters indicate statistically significant differences within each fraction at *p* < 0.05, as determined by one-way ANOVA followed by Tukey’s post hoc test

To gain deeper insight into the CW composition of WT and CWP strains, microalgal dry biomass was subjected to extraction using alcohol and chloroform to eliminate the alcohol-soluble fraction, resulting in the alcohol-insoluble residue (AIR), which was subsequently enzymatically de-starched (Fig. 5a). De-starched AIR corresponded to about 80% of the initial dry biomass of the wild type and of lines CWP3 and 6, but was significantly less in CWP1, 2, 5 (about 60%) and CWP4 (about 50%). To further analyze the chemical composition of the CW of the selected mutants, the polysaccharides in the AIR were hydrolyzed with trifluoroacetic acid (TFA) and subsequently monosaccharide composition of the hydrolysate was analyzed by HPAEC-PAD. Approximately 40% of the AIR was not hydrolyzed by TFA, representing the most resistant fraction of the CW; this fraction displayed a similar relative abundance across all strains (Fig. 5b). The most abundant monosaccharides present in the hydrolyzed fraction of the AIR of the WT strain were rhamnose and galactose, accounting for 35% and 22% of the total monosaccharides, respectively, followed by glucosamine (12%) and glucose (10%); minor components included arabinose, xylose, mannose, and fucose. These data are consistent with the relative abundance of CW monosaccharides reported by other authors [35, 55]. In contrast, all CWP strains showed a reduced amount of rhamnose and glucosamine and an increased amount of galacturonic acid, along with variations in the relative abundance of galactose, glucose, and xylose compared to the WT strain (Fig. 5c).

To determine whether the changes in CW content and composition affected resistance to mechanical disruption in different strains, we performed wet milling with glass beads on both WT and mutant dry biomass, followed by measurement of the proteins released into the supernatant, in order to evaluate the effectiveness of the CW disruption. As illustrated in Fig. 5d, the mechanical treatment resulted in low levels of released proteins across all strains, indicating that the strength of the CW remained largely unaffected in all CWP strains.

### Isolation of CFW, CW-weakened strains of C. vulgaris

The results presented in the earlier sections demonstrated that mutagenesis combined with FACS-based selection was successful in isolating *Cv* strains with modified CW permeability to specific probes. However, none of the CWP strains exhibited improved biomass extraction efficiency. It was hypothesized that a reduction in CW material and/or structural changes in CW polymers, which likely occurred in CWP lines (as illustrated in Fig. 5), merely enhanced the permeability of the CW to small molecules, increasing the affinity of the probes to the cells; yet, the CW remained robust enough to withstand the tested disruption and extraction methods. In addition, it is important to consider that the biogenesis of a dynamic and complex structure like the CW could be regulated by multiple gene families. Therefore, several rounds of mutagenesis and selection might be needed to identify mutant lines with significant changes in CW strength.

To this aim, we implemented a FACS-based screen focusing on lines showing reduced staining with Calcofluor White (Cf), a dye that typically binds to cellulose and chitin [56]. The CWP1 strain, which exhibited noticeable changes in both structure and composition of the CW (Figs. 3, 4, 5), was selected as the parental genotype for a second round of mutagenesis. As depicted in Figure S7, CWP1 cells stained with Cf exhibited strong fluorescence under blue light, indicating the presence of cellulose or chitin in their CWs, akin to other algae [57]. After treatment with an enzymatic cocktail containing lysozyme, chitinase, and sulfatase (refer to treatment E1, Figure S2) plus 1% cellulase, the cells showed reduced fluorescence upon Cf staining, implying the removal of the cellulose/chitin layer surrounding these cells.

These two *Cv* populations, distinguishable by their different CW properties via Cf staining, were used to validate the FACS-based cell sorting method, similar to the previous experiments (Figure S3). The CWP1 populations were treated with EMS (1.6–3.4%) to create a mutant library. The next step involved staining the algal cells with Cf and using flow cytometry to sort out nonfluorescent cells (Figure S8). In this case, we selected events contained in the P4 region, corresponding to living cells with low fluorescence to the dye, and then screened again for cells with high SSC-A signal and low Cf signal, suggesting alterations in CW composition (P6 region). This population, corresponding to approx. 2600 events, was collected and cultured at low density on BG-11 agar plates, with single colonies emerging after three weeks. After eliminating mutants with poor growth, 75 colonies were transferred to liquid medium in microtiter plates.

Bead milling was applied to the biomass of mutant strains to assess their CW strength. This mechanical treatment effectively causes significant damage to the *Cv* CWs [58], and the consistent release of proteins into the supernatant (Figure S9) makes it an efficient and rapid screening method. As shown in Figure S10B, bead-beating identified six lines with the highest protein release. Of these, two lines with the greatest protein extractability were selected for further analysis and named CFW1 and CFW2.

**Fig. 6:**
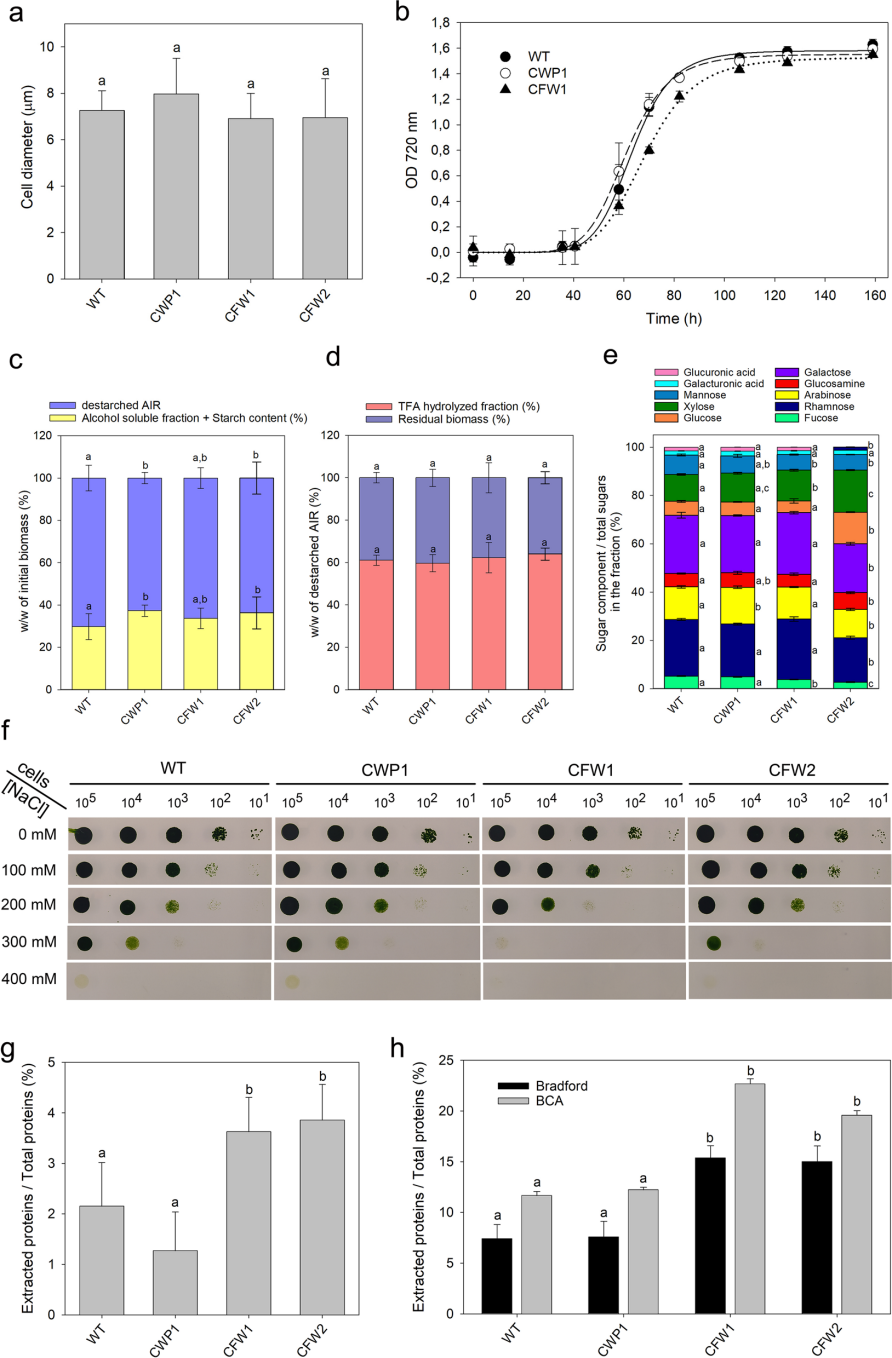
Characterization of CFW mutant in *C. vulgaris.* **a** The average cell size of WT and mutants, which showed no significant differences (ANOVA followed by Tukey’s post hoc test at a significance level of *p* < 0.05). **b** Growth curves under autotrophic conditions. *Cv* WT and cell wall mutants growth was assessed by daily measuring the OD at 720 nm. Experiments were conducted in 100 mL cylinders (MultiCultivator, PSI) in a semi-batch system fed with air/CO_2_ mix, illuminated with 80 μmol photons m^−2^ s^−1^, at 24 °C. Data are shown as mean ± SD, and are representative of two independent experiments. Initial inoculum was 5·10^5^ cells mL^−1^. **c**–**e** Analysis of cell wall composition. Percentage weight of the various fractions compared to the initial biomass weight **c** and to the  alcohol-insoluble residue (AIR) weight **d**. **e** Percentage weight of the sugar component relative to the total sugars weight in the fraction. The bars represent the mean ± SD (*n* = 3). Different letters indicate statistically significant differences within each fraction at *p* < 0.05 (one-way ANOVA followed by Tukey’s post hoc test). **f** Salt stress effect on the growth of *Cv* WT and mutant lines were tested by spotting 10 μL serial decimal dilutions onto TAP-agar plates supplemented with 0–500 mM NaCl. The number of cells spotted is indicated on the upper border. Plates were illuminated with 80 μmol photons m^−2^ s^−1^, photoperiod of 16/8 h light/dark, 24 °C. **g**, **h** Protein extraction efficiency from algal biomass, disrupted with either a bead beater with glass beads **g** or sonication **h**, the protein content in the supernatant was measured relative to the total biomass protein. Protein levels are represented by black bars (Bradford methods) and gray bars (BCA methods). Different letters indicate statistically significant differences within each fraction at *p* < 0.05, as determined by one-way ANOVA followed by Tukey’s post hoc test

No significant differences were observed in cell size or photoautotrophic growth rates among the CFW lines as compared to the parental line (Fig. 6a, b). Characterization of the mutant CW revealed that the relative abundance of AIR and the more rigid CW fraction was similar across all genotypes. Analysis of the monosaccharide components of the de-starched AIR obtained after TFA hydrolysis indicated minor changes, with the CFW2 line showing reduced rhamnose and galactose content, offset by higher levels of xylose and glucose (Fig. 6c-e). The permeability of CFW cells to the dyes FDA and EB remained similar to that of the parental line (Figure S11).

To further evaluate whether CW strength was reduced, cells were plated on agar with increasing concentrations of NaCl. The CW is essential for maintaining cell morphology, integrity, and the ability to respond to environmental stresses. Salt stress, in particular, exacerbates hyperosmotic stress in cells with weakened CW integrity [59]. The results (Fig. 6f) indicated that higher NaCl concentrations significantly affected growth in all genotypes. However, the effect was more severe in the CFW lines compared to the parental strains, with the former exhibiting lower survival rates at NaCl concentrations of 300 mM and above. To assess their susceptibility to cell wall disruption, different mechanical treatments were applied to the biomass from both CFW strains, specifically 2 min of wet milling with glass beads or 30 min of sonication. In both cases, the amount of protein released was significantly higher in the CFW strains than in the control genotypes (Fig. 6g, h).

In summary, the extraction efficiency of intracellular compounds was enhanced in the CFW mutants without negatively impacting their growth rate. This validates the effectiveness of FACS-based high-throughput screening in identifying strains with multiple advantageous traits for industrial applications.

## Discussion

### Biotechnological importance of CW-weakened strains

The genus *Chlorella* has become noteworthy for its robust strains that accumulate biomass effectively, supporting sustainable industrial production of high-value products and biofuels [60]. However, the thick and rigid CW poses significant challenges in extracting intracellular compounds and limit their bioavailability for human and animal consumption when used whole as nutraceuticals or health food additives [61]. Harsh physical/chemical extraction methods like high-pressure homogenization, bead milling, pH-dependent hydrolysis, and autoclaving, alongside milder enzymatic digestion, have been employed to break the CW barrier [62]. Despite their use, these techniques are often inefficient, costly, and can lead to the oxidation and degradation of valuable biochemicals due to exposure to atmospheric oxygen. Thus, developing CW-weakened mutant strains offers a promising strategy to improve the extraction quality from *Chlorella* biomass while reducing cell disruption and processing costs. The mutants produced in this study initiate this approach.

### Screening of microalgal mutants for altered CW

The properties of the CW are important in algal biotechnology; however, engineering the CW in algae has been successful in only a few instances [39]. This is mainly due to the lack of reliable transformation protocols for most nonmodel species and the necessity for high-throughput screening techniques. Chemical mutagenesis, which induces high-frequency base-pair substitutions within the genome, has been used to develop CW-deficient mutant strains in bacteria, yeasts, and plants. In microalgae, this method has produced several CW-deficient mutants of *C. reinhardtii* [36]. TEM analysis has helped categorize these mutants into three classes based on their ultrastructural features: class A, where the wall is not attached to the plasma membrane; class B, where the wall is anchored to the plasmalemma and produced in amounts similar to the wild-type strain; and class C, where the wall is significantly reduced or absent [37]. According to these criteria, all the *Cv* mutants generated in this study likely belong to class B.

Traditionally, screening for CW-deficient algal strains involved microscopic observation [36, 63], which is highly time intensive. An alternative approach could involve targeting algal CW glycans with specific antibodies developed against plant CW epitopes [63], although this might be complicated by the unique composition of algal CWs. Moreover, a reduction of a specific epitope in the CW does not necessarily imply that the CW of the mutant has reduced mechanical resistance. The mutant screening approach used in this study, which is based on flow cytometry, offers higher throughput than traditional methods and allows the selection of strains with impaired CW properties, regardless the structural components that are altered by the mutations. A recent study demonstrated a successful FACS-based pipeline that identified CW-deficient mutants of the green alga *Haematococcus pluvialis* [38].

The crucial step in developing a FACS-based screening procedure is establishing a protocol to distinguish cells with altered CW while minimizing false-positive results. To achieve this, we assessed the permeability of *Cv* CW to specific dyes in populations with compromised CW integrity. By precisely adjusting the concentration of fluorescent probes, we validated the flow cytometry-based cell sorting method. This approach allows for the differentiation of cell groups based on their CW permeability, as indicated by EB and FDA staining (Figure S1-4).

Although *Cv* is among the most extensively studied microalgae, detailed information on its CW structure and composition remains incomplete. Early studies on the CW biochemical composition of members of the genus *Chlorella* [64] have been complicated by taxonomic revisions, which challenge the direct comparison of literature data, and by ample variation in the conditions and stage of growth analyzed and in the protocols of CW isolation and biochemical characterization. Ultrastructural analysis reveals that the development of the CW in *Cv* is influenced by its growth phase: cells initially have a thin and fragile CW composed of a single microfibrillar layer during the early growth phase, which quickly evolves into a three-layer structure [41]. Its thickness increases to approximately 80 nm during the exponential phase and 115 nm in the stationary phase. CW thickness measured in this study is consistent with the literature, which reports values ranging from 80 to 150 nm [44]. Notably, strains CWP1 and CWP5 displayed thicker CWs than other strains (Fig. 3), which might suggest increased resistance during downstream processing. However, this was not observed; all strains demonstrated similar mechanical resistance (Fig. 5), implying that CW thickness does not correlate to its robustness. This finding aligns with observations of lipid extractability in *Cv* biomass, which was greater in the stationary phase compared to the exponential phase [65], indicating a higher CW vulnerability during the latter stage. A thicker CW such as that observed in CWP1 and CWP5 (Fig. 3) might indeed form a more flexible, less compact barrier, that is more prone to swelling and more permeable to small molecules, like the fluorescent dyes used in the FACS-based screening, but with intact resistance to mechanical disruption.

In different species (such as *Chlorella spp., Scenedesmus spp.,* etc.) with different CW thicknesses, the extractability of intracellular content depends not only on the thickness of the CW but also on its composition and architecture [66]. A previous extensive characterization of a wide range of *Cv* strains indicated that a distinctive trait of this species is the presence of a glucosamine-rich CW, which is stainable with red ruthenium [55], indicating the presence of chitin-like polysaccharides as well as uronic acid-rich polysaccharides. A study implementing stepwise degradation of the *Cv* CW identified chitin- or chitosan-like polymers due to glucosamine presence in strong alkali extracts, while the detection of glucose in strong acidic extracts suggested cellulose as a component [45]. It can be hypothesized that the CW of *Cv* comprises both chitin-like and cellulose- and glucan polysaccharides, representing the more rigid polysaccharidic components of the CW, providing resistance to tension and to mechanical disruption, as well a less rigid matrix component. The latter is possibly represented by galactans and pectin-like polymers containing rhamnose, as also previously proposed [42], although to date no structural characterization of these polysaccharides in *Cv* is available. However, pretreatments with rhamnohydrolases or galactanases were reported to increase the release of carbon and nitrogen upon high-pressure homogenization treatment of *Cv* biomass, supporting the hypothesis that galactose- and rhamnose-rich polysaccharides contribute to the mechanical strength of the CW of *Cv* [42]. It must be noted that reports about the monosaccharide composition of *Cv* CW vary significantly. Some studies [43] primarily noted rhamnose, arabinose, and glucose, while others [64] reported high levels of galactose and rhamnose. These discrepancies may be attributed to differences in taxonomic classification, or physiological variations resulting from distinct growth conditions or cellular states, which could influence CW biochemical composition. Our findings support the view that the *Cv* CW WT strain has a highly recalcitrant and rigid CW structure. Approximately, 40% w/w of the destarched AIR was not hydrolyzed by the acid treatment employed, underscoring the CW’s robust resistance. Among the hydrolyzed fraction, rhamnose and galactose were the most abundant monosaccharides, accounting for 35% and 22% of the total sugars, respectively. The presence of glucosamine (12%) confirms the contribution of chitin-like polymers to CW rigidity, while glucose (10%) and minor sugars, such as arabinose, xylose, mannose, and fucose add to the complexity of the CW, in agreement with previous studies [35, 55]. We also support the results of studies on the CW of *Chlorella sorokiniana*, which identified rhamnose as the primary contributor to CW resistance. These findings collectively highlight the interplay of polysaccharides, including chitin-like polymers, rhamnose-rich components, and other structural sugars, in conferring the observed mechanical strength and resilience of the *Cv* CW [42, 45].

FTIR spectroscopy is not sensitive enough to provide a detailed profile of the sugar composition, but it suggests a role of β-glucans, particularly β−1,6 glucans, in the architecture of the CW. Consistent with TEM images, FTIR results suggest that the increased permeability of the CW may be attributed not so much to a different biochemical composition, but rather to a looser structure. This loosening of the CW structure may be related to a relatively lower presence of β−1,6 glucans and, to a lesser extent, β−1,4 glucans, when combined with a relative increase in β−1,3 glucans.

Notably, our results indicate that some of the CWP mutants have a reduced amount of TFA-resistant AIR fraction (Fig. 5), which might have led to an increased permeability of the fluorescent dye during the selection process. This fraction is mostly represented by chitin and, possibly, cellulose-like polymers. Monosaccharide composition of the TFA-sensitive fraction of the AIR in the CWP mutants appears to be only slightly altered (Fig. 5), as compared to the WT parental strain. Notably, CWP strains showed reductions in rhamnose and glucosamine content, accompanied by increases in galacturonic acid, galactose, glucose, and xylose, depending on the strain. These compositional changes suggest a reorganization of CW polysaccharides, potentially favoring components that enhance permeability. For instance, the increased galacturonic acid content might indicate a shift toward pectin-like polymers, which, being less rigid, may enhance probe affinity during permeability tests. In addition, some evidence suggests the presence of sporopollenin, a highly resistant polymer of mostly aliphatic nature, in the CW of related species such as *C. protothecoides* [67]. This latter component, which is resistant to TFA hydrolysis, might also be a factor of the robustness of the *Cv* CW. While the observed reduction of the rigid fraction in CWP mutants may have enhanced CW permeability, it was insufficient to significantly improve biomass extractability. Structural polymers such as chitin, cellulose-like components, and sporopollenin appear to maintain substantial mechanical resistance in the CW. Consistently low protein release levels across strains after wet milling (Fig. 5) confirm that overall CW mechanical resistance remained unaffected. These findings suggest that CW modifications in CWP strains primarily enhance permeability to small molecules without reducing CW strength. The increased CW porosity, likely due to reductions in rhamnose and glucosamine, facilitates probe diffusion, while the conserved rigid fraction ensures mechanical robustness. The dynamic nature of CW biogenesis, regulated by multiple gene families and feedback mechanisms, further complicates these observations.

The limited improvement in biomass extraction efficiency in CWP strains indicates that a single round of mutagenesis was insufficient to achieve significant weakening of the CW. Consequently, an additional round of mutagenesis and screening was performed to isolate CFW mutants with CWs exhibiting reduced mechanical resistance.

### The extraction efficiency was significantly enhanced in the CFW lines

Two rounds of chemical mutagenesis led to the isolation of two CFW lines, which exhibited a twofold increase in biomass extraction efficiency as compared to the parental line. However, it is noteworthy that despite the 30 min sonication treatment—a relatively harsh extraction method—less than a quarter of the total proteins in the biomass were extracted (Fig. 6g, h). This indicates that the CW remains robust in the selected CFW mutants.

In *Chlamydomonas*, a study [36] identified CW-deficient mutants, including UVM4, which displayed increased sensitivity to shear stress compared to the WT strain [68]. Through intercrossing individual mutants in all combinations, the authors found that most of their mutants resulted from single-gene changes. The biogenesis of CW involves complex metabolic processes, such that mutations affecting various cellular, physiological, and developmental functions can potentially influence CW structure. These processes may include the biosynthesis of sugar substrates and polysaccharides, the activity of the cytoskeleton and secretory pathways, as well as various signaling pathways.

The CWP and CFW strains generated in this study could be a result of either single- or multiple-gene mutations. However, due to the lack of sexual reproduction in *Cv*, identifying the mutations responsible for these phenotypes would only be approachable by whole-genome sequencing. Our results indicate that two rounds of mutagenesis and FACS selection were required to impact CW mechanical resistance in *Cv*, suggesting a higher level of complexity in the cellular targets of this species compared to *Chlamydomonas*. Therefore, isolating mutant lines undergoing significant alteration of a dynamic and complex carbohydrate-rich structure like the CW will likely demand multiple cycles of mutagenesis and selection to achieve substantial advancements, as demonstrated for other metabolic targets [69]. Furthermore, CW weakening induced by mutagenesis may have activated compensatory mechanisms that reshape the CW architecture to counteract cell lysis, as previously observed in yeast [70] and in the *C. reinhardtii* CW15 strain [71]. It is also plausible that weak CW-deficient mutants could have emerged from chemical mutagenesis; however, negative selection due to increased sensitivity to osmotic shock or shear stress during growth in photobioreactors or during FACS could have hindered their identification.

## Supplementary Information

Supplementary material 1. Figure S1. Mechanism of action of fluorescent probes used and schematic representation of the screening process. Figure S2. Dependence of the retention of fluorescent probe on cell vitality and cell wall integrity. Figure S3. Setup of a screening strategy for isolating *C. vulgaris* mutants with increased cell wall permeability (CWP), utilizing flow cytometry. Figure S4. Example of sorting of EMS-mutagenized cell populations based on FDA and EB fluorescence. Figure S5. Summary table of parameters for selecting the 6 putative CWP mutants. Figure S6. FTIR spectroscopy of cell wall components. Figure S7. Fluorescence emission of CWP1 cells stained with Calcofluor White. Figure S8. Sorting of EMS-mutagenized CWP1 cells based on Cf fluorescence. Figure S9. Setup of a bead-beating method for protein extraction from Cv biomass. Figure S10. Characterization of mechanical resistance in Cv lines selected for lower Cf binding affinity. Figure S11. Permeability of CWP1 and CFW cells to fluorescent probes. Table S1. Data output from sorting performed by the FACSAria Fusion, for isolating CWP mutants.

## Data Availability

No datasets were generated or analysed during the current study.
