# Supplementary material for: Chlorella vulgaris mutants with altered cell walls show increased permeability and enhanced extractability of intracellular molecules
Source: Biotechnol Biofuels Bioprod. 2025 Jun 5;18:59. doi: 10.1186/s13068-025-02663-0 (PMC12142970; doi:10.1186/s13068-025-02663-0)
Supplement: Supplementary file 1 — Supplementary material 1. Figure S1. Mechanism of action of fluorescent probes used and schematic representation of the screening process. Figure S2. Dependence of the retention of fluorescent probe on cell vitality and cell wall integrity. Figure S3. Setup of a screening strategy for isolating C. vulgaris mutants with increased cell wall permeability (CWP), utilizing flow cytometry. Figure S4. Example of sorting of EMS-mutagenized cell populations based on FDA and EB fluorescence. Figure S5. Summary table of parameters for selecting the 6 putative CWP mutants. Figure S6. FTIR spectroscopy of cell wall components. Figure S7. Fluorescence emission of CWP1 cells stained with Calcofluor White. Figure S8. Sorting of EMS-mutagenized CWP1 cells based on Cf fluorescence. Figure S9. Setup of a bead-beating method for protein extraction from Cv biomass. Figure S10. Characterization of mechanical resistance in Cv lines selected for lower Cf binding affinity. Figure S11. Permeability of CWP1 and CFW cells to fluorescent probes. Table S1. Data output from sorting performed by the FACSAria Fusion, for isolating CWP mutants. [file 13068_2025_2663_MOESM1_ESM.docx]

**Additional results**

**Figure S1**

**
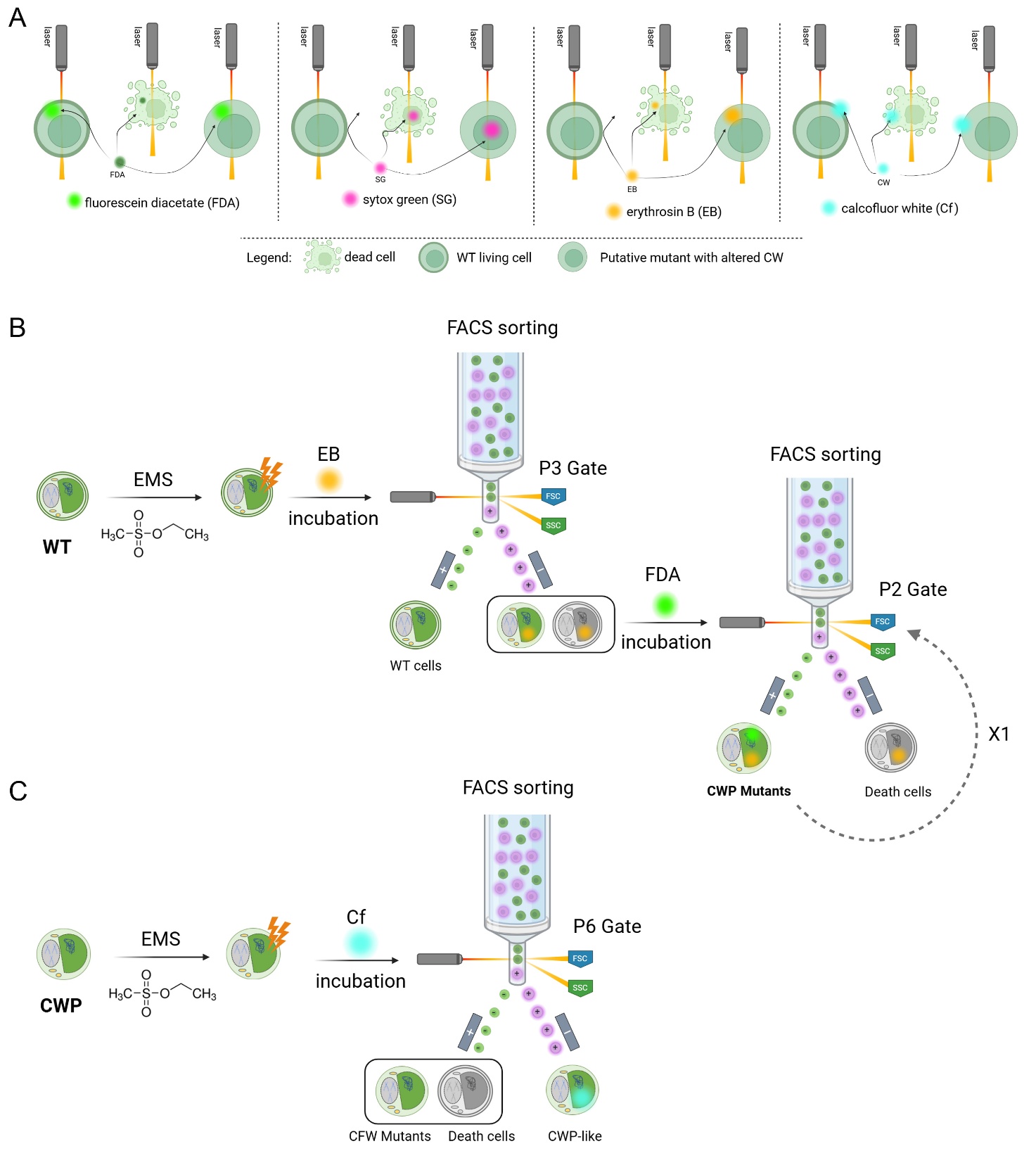
**

**Figure S1. Mechanism of action of fluorescent probes used and schematic representation of the screening process.** (A) Fluorescein diacetate (FDA) is a non-polar, non-fluorescent probe that can freely enter cells. Once inside, FDA is metabolized by non-specific esterases, resulting in the production of fluorescein and acetate. Fluorescein is fluorescent and charged, rendering it unable to cross the cell membrane in living cells. Consequently, the intensity of FDA fluorescence can serve as an indicator of metabolic activity and indirectly as a viability marker. SYTOX Green (SG) is a DNA-staining dye that cannot cross the intact cell wall/membrane barrier. Flow cytometry enables the distinction between live and dead cells by producing an increased fluorescent signal upon binding to DNA. Erythrosin B (EB) is a polar molecule impermeable to cell membranes, allowing it to penetrate only dead cells or those with a damaged/altered cell wall (CW). Calcofluor white (Cf) binds to β-1,3- and β-1,4-linked polysaccharides, such as those found in cellulose and chitin, present in microalgal cell walls. (B,C) Schematic workflow of the screening and selection process for microalgal cells, which involves (i) chemical mutagenesis with EMS, (ii) staining of target cells with EB+FDA (panel B) or of CW components with Cf (panel C), (iii) screening and sorting of cells based on altered staining level, (iv) recovery of cells with desired features, which in the case of (B) were reintroduced into iterative selection, and (v) growth of selected cells.

**Figure S2**


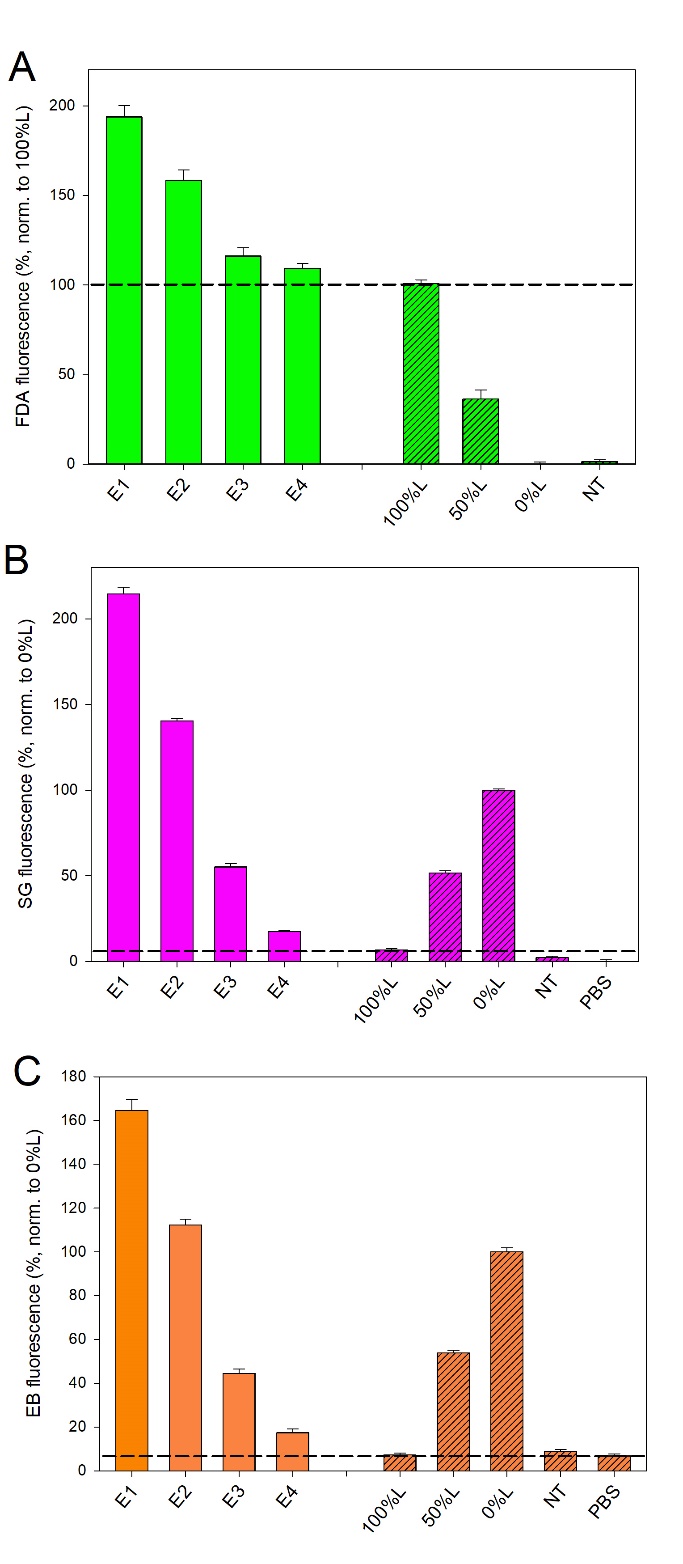


**Figure S2. Dependence of the retention of fluorescent probe on cell vitality and cell wall integrity.** *Cv* WT cells (5·10^6^ cells) were treated with various enzymes combinations to weaken their cell wall and alter permeability: treatment E1 used lysozyme + chitinase + sulfatase; treatment E2 used lysozyme; treatment E3 used cellulase; treatment E4 used chitinase. Internal controls included heat-inactivated WT cells, used to prepare samples with different proportions of living cells: 100%L (5·10^6^ living cells), 50%L (2.5·10^6^ living and 2.5·10^6^ dead cells), and 0%L (5·10^6^ dead cells). An additional control, not treated (NT), was prepared by incubating 5 × 10⁶ cells only with enzymatic buffer, and PBS buffer was used as a blank. All samples were stained with (A) Fluorescein diacetate (FDA) to assess cell viability, (Bb) SYTOX Green (SG) or (C) erythrosin B (EB) to evaluate cell permeability. Fluorescence was measured using a plate reader, and results were normalized to internal controls with the highest fluorescence yield: 100%L for FDA, 0%L for EB and SG. The threshold for increased permeability (100%L) was indicated by the black dotted line in panels b and c. Data are expressed as mean ± SD, n = 3.

**Figure S3**


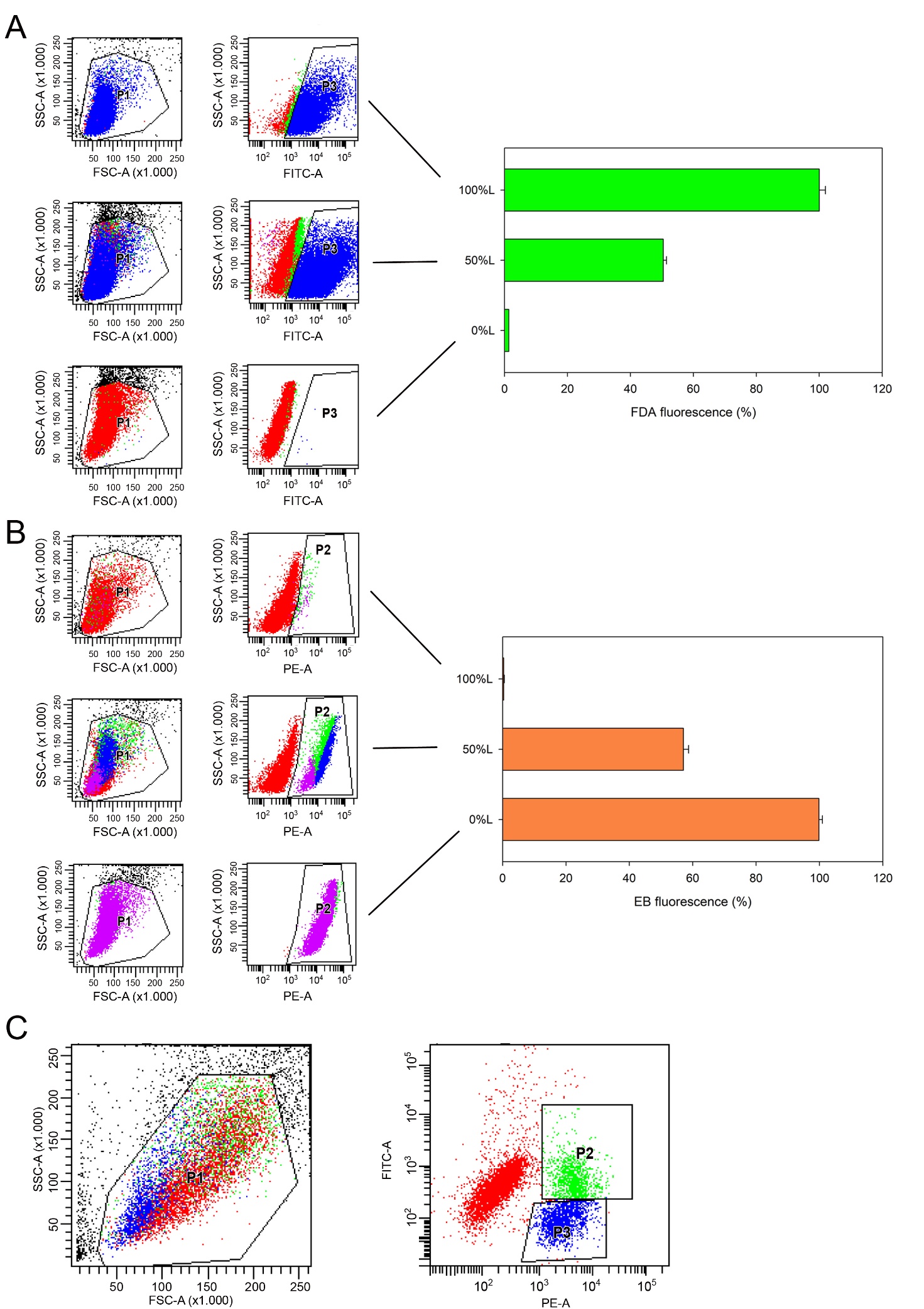


**Figure S3. Setup of a screening strategy for isolating *C. vulgaris* mutants with increased cell wall permeability (CWP), utilizing flow cytometry.** (A, B) Dot plots of algal cells were generated using flow cytometry from populations with varying proportions of living cells: 100%L, 50%L and 0%L (refer to Figure S2). The same sample was stained with (A) Fluorescein diacetate (FDA) or (B) erythrosin B (EB) and analyzed with FACSAria Fusion Cell analyzer with fluorescein isothiocyanate (FITC) and phycoerythrin (PE) channel, respectively. In all the analyses, two dot plots were produced: (*left graph*) distribution of cell granularity and size as detected via forward scatter (FSC) and side scatter (SSC); (*right graph*) distribution of cells based on SSC and fluorescence detection via the FITC or PE channels. The gating was set as follows: P1 for the total selected cells; P3 for cells with a high FDA signal, indicating living cells; and P2 for cells with permeable cell walls, characterized by high EB signals. (C) In addition, *Cv* WT cells treated with E1 enzyme mix were stained with both FDA and EB and analyzed using simultaneously FITC and PE channels for fluorescence detection of FDA and EB, respectively. In this case, an FSC-SSC dot plot (left) shows cell features of the sample, and a dot plot of FITC-PE channel detection (right) shows cell distribution based on FDA and PE detection in the sample. The gating was set as follows: P1 for total selected cells; P3 for cells with high EB and low FDA signals, indicating dead cells; and P2 for cells exhibiting high signal for both EB and FDA, suggesting increased cell wall permeability.

**Figure S4**


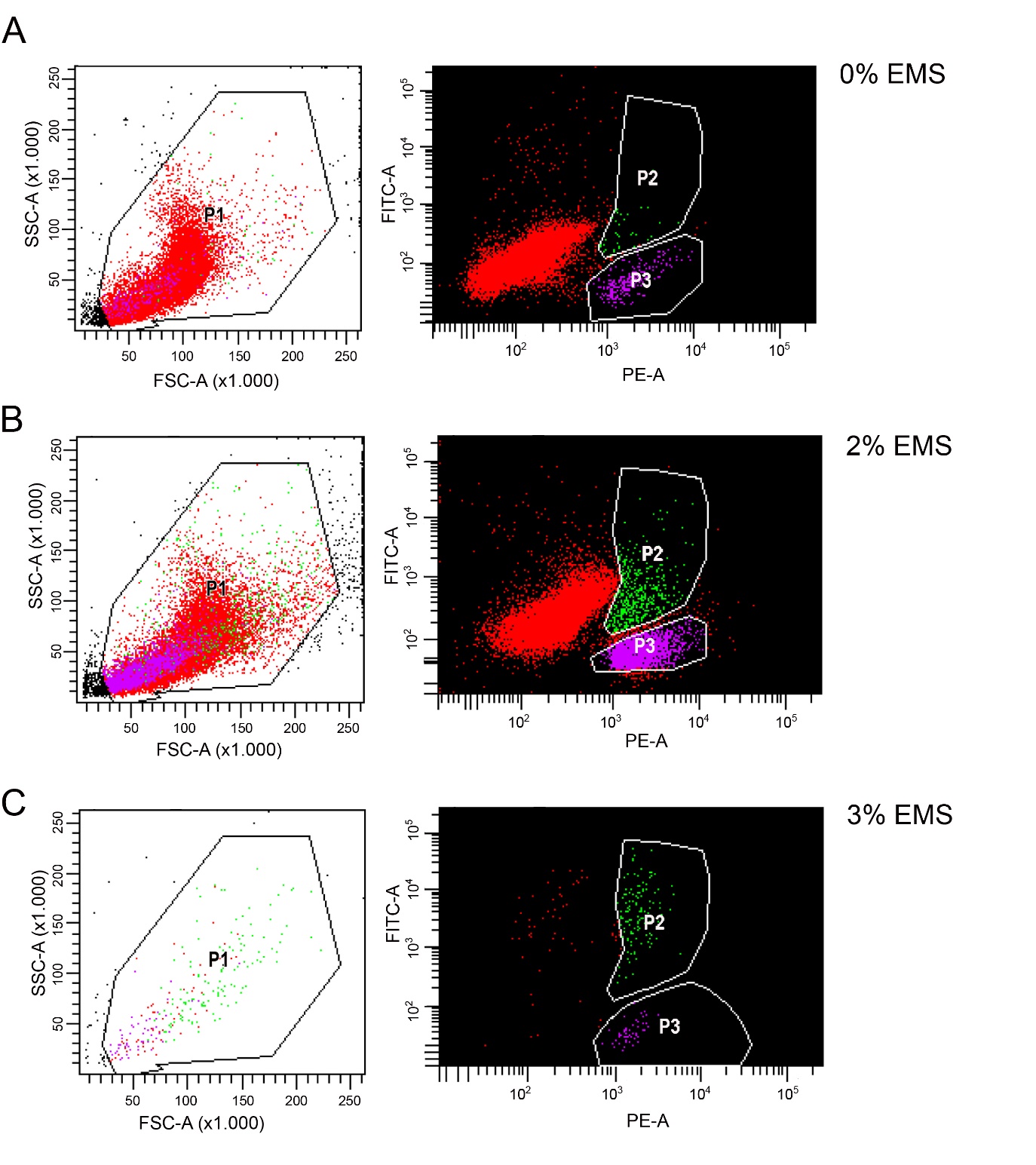


**Figure S4. Example of sorting of EMS-mutagenized cell populations based on FDA and EB fluorescence.** Dot plots of algal cells were generated using flow cytometry from populations mutagenized with EMS: (A) untreated WT cells, or (B) treated with 2% EMS. The cells were stained with both the selective probes fluorescein diacetate (FDA) and erythrosin B (EB), and high-fluorescent cells were sorted out using the FACSAria Fusion Cell sorter (refer to Figure S2). Two dot plots were produced for each sorting: (*left graph*) distribution of cell granularity and size as detected via forward scatter (FSC) and side scatter (SSC); (*right graph*) distribution of cells based on fluorescence detection via the phycoerythrin (PE) (for EB) or fluorescein isothiocyanate (FITC) (for FDA) channels. The gating was set as follows: P1 for total selected cells; P3 for cells with high EB and low FDA signals, indicating dead cells; and P2 for cells exhibiting high signal for both EB and FDA, suggesting increased cell wall permeability.

**Figure S5**

**Figure S5. Summary table of parameters for selecting the 6 putative CWP mutants**. This table presents the following parameters: cell density (cells mL^-1^, x10^8^) after 3, 6 and 9 days of growth (initial inoculum: 5·10^5^ cells mL^-1^); permeability to the dyes fluorescein diacetate (FDA), SYTOX Green (SG) and erythrosin B (EB), measured at day 3, with fluorescence values normalized against internal controls: WT living cells (100%L) for FDA, WT dead cells (0%L) for EB and SG (refer to Figures S1, S2). The assessment for FDA permeability was categorized as follows: low (1-2 times higher than the control sample), medium (2-3 times higher than the control sample), and high (> 3 times higher than the control sample). For the fluorescent dyes EB and SG, the permeability values are classified as follows: low (≤ 25% of the control sample), medium (25-50% of the control sample), and high (> 50% of the control sample). The color intensity of each box corresponds to the variation compared to the control. The 6 selected lines are marked with the red tick.

**Figure S6**


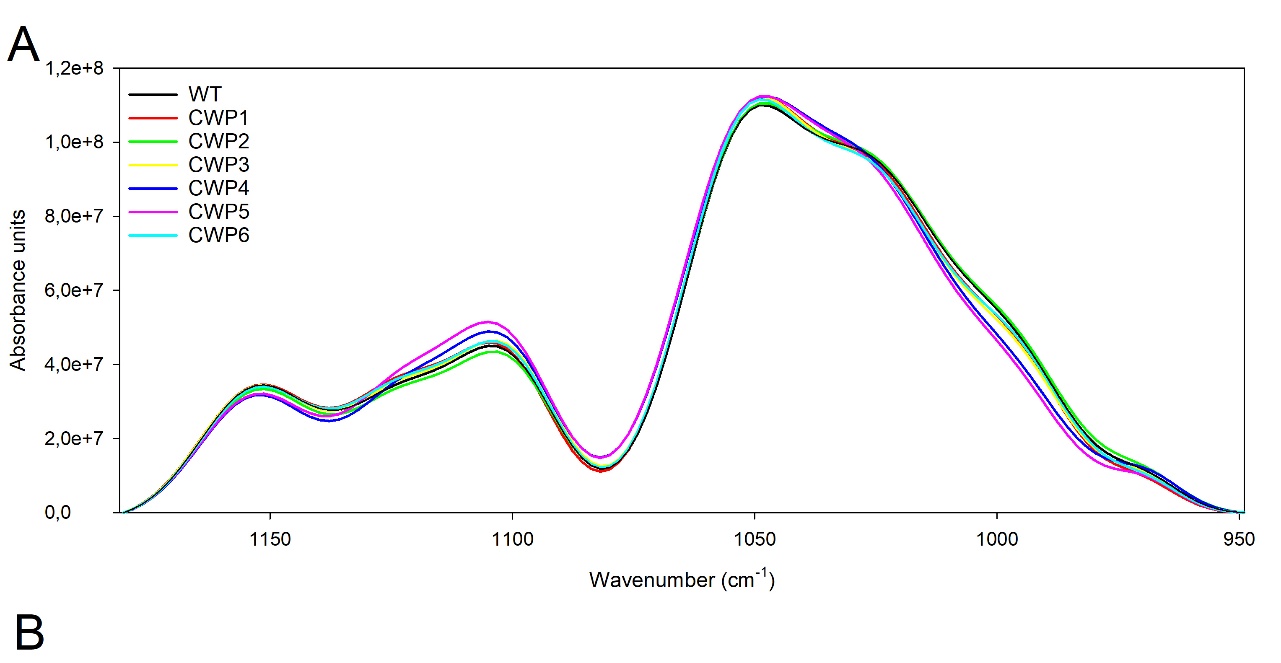

**Figure S6. FTIR spectroscopy of cell wall components.** (A) Reconstructed spectra of the WT and CWP mutant lines in the 1180–950 cm^-1^ range, obtained as the area-normalized sum of all the fitted Gaussian bands (see Figure 4), except for the one at 1080 cm^-1^. (B) Positions of the absorption bands in the 1180–950 cm^-1^ range, as identified from the minima of the second derivative of the spectra, along with their corresponding assignments as determined from [54 and references therein].

**Figure S7**

**
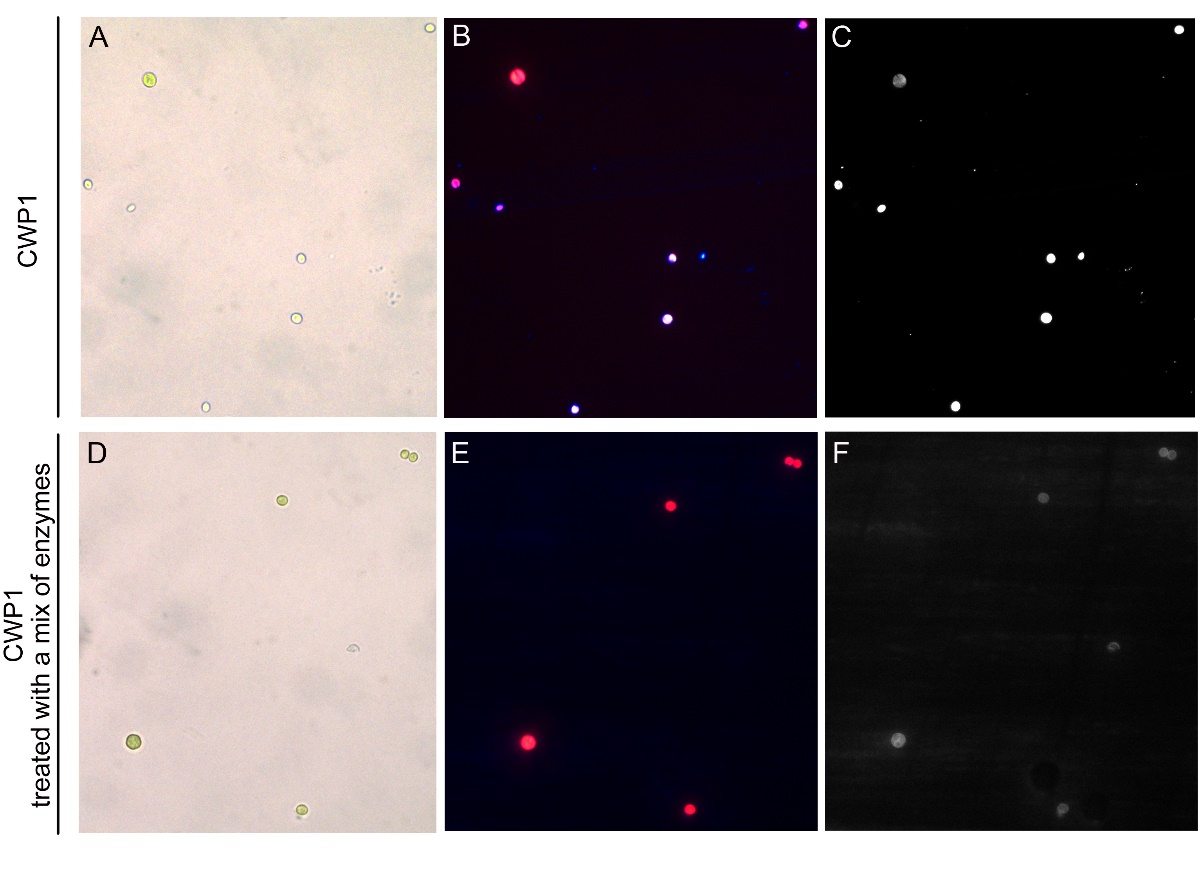
**

**Figure S7. Fluorescence emission of CWP1 cells stained with Calcofluor White.** (A, D) Light microscopy images (×400) of *Cv* CWP1 cells. (B, E) Chlorophyll fluorescence images of the same cells (λ_exc_ = 330-385 nm, λ_emis_ > 420 nm). (C, F) cells after staining with Calcofluor White (Cf, detection in the blue channel). Panels control (A-C) represent control cells, while panels (D-F) show cells treated with a mix of cell-wall degrading enzymes (lysozyme, chitinase, sulfatase, cellulase). The fluorescence intensity of Cf in enzymatically-treated cells was 22 ± 7% compared to untreated cells (n = 7).

**Figure S8**

**
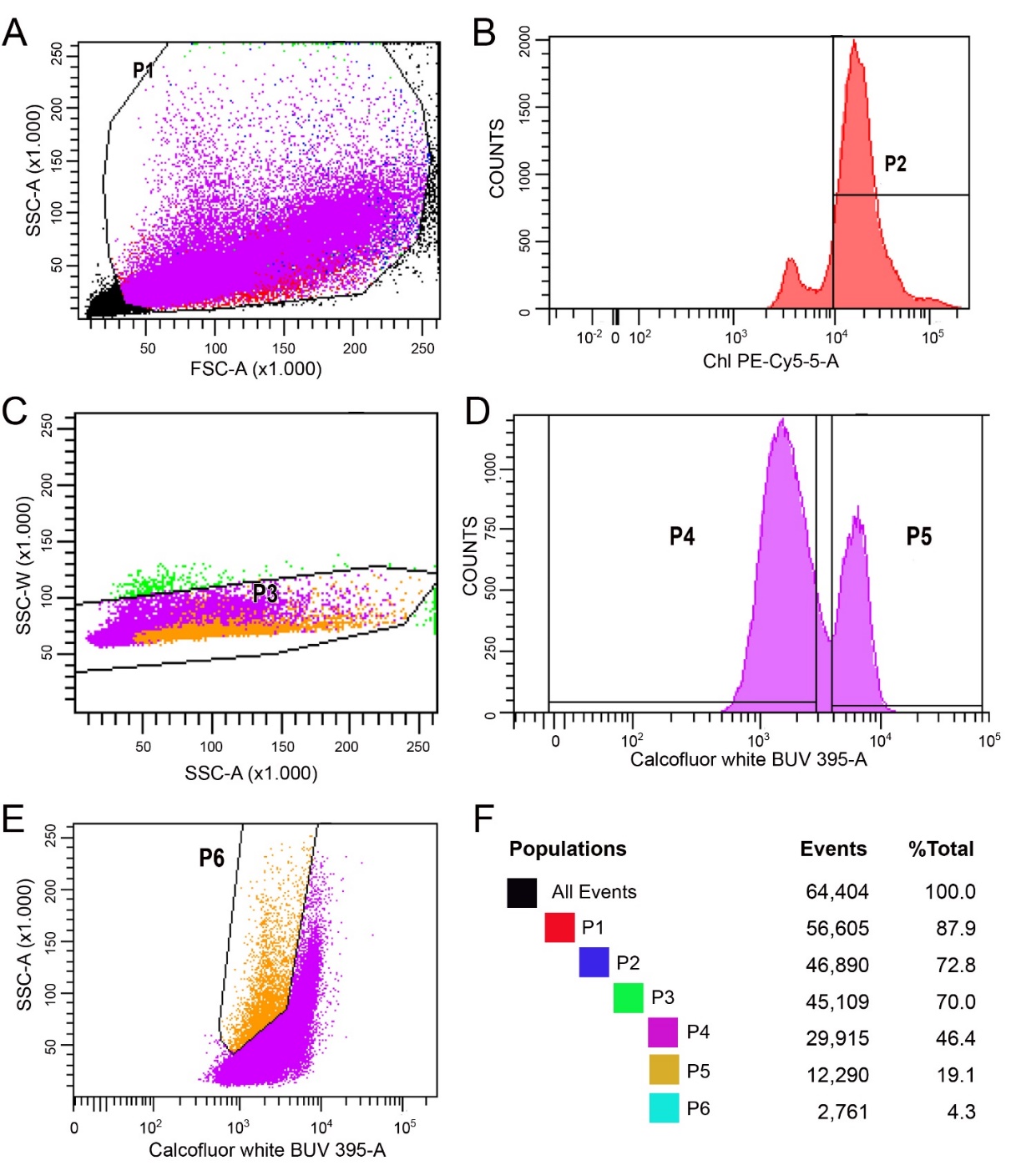
**

**Figure S8. Sorting of EMS-mutagenized CWP1 cells based on Cf fluorescence.** Dot plots were generated using flow cytometry from a population of CWP1 cells mutagenized with 2% EMS. The cells were stained with the selective probe Calcofluor white (Cf), which specifically binds to β-1,3 and β-1,4 polysaccharides, such as those found in cellulose and chitin. Low-fluorescent cells were sorted using the FACSAria Fusion Cell sorter (refer to Figure S2). Different plots were produced for each sorting, including: (A) the distribution of cell granularity and size, as detected through forward scatter (FSC) and side scatter (SSC); (B) the distribution of cells based on chlorophyll fluorescence yield; (C) the side scatter width (SSC-W) and amplitude (SSC-A), which provide insights into the internal complexity (granularity) of cells; and (D,E) cell counts and dot plots of Cf stained cells. In the right-hand graphs, the gating was set as follows: P1 for the total selected cells; P2 for cells exhibiting high chlorophyll fluorescence; P3 for unaggregated cells; P4 for cells showing low Cf signals, indicating a low binding affinity for the dye while P5 show cells with opposite behavior; and P6 for cells with high SSC-A signal and low Cf signal, suggesting alterations in cell wall composition.

**Figure S9**

**
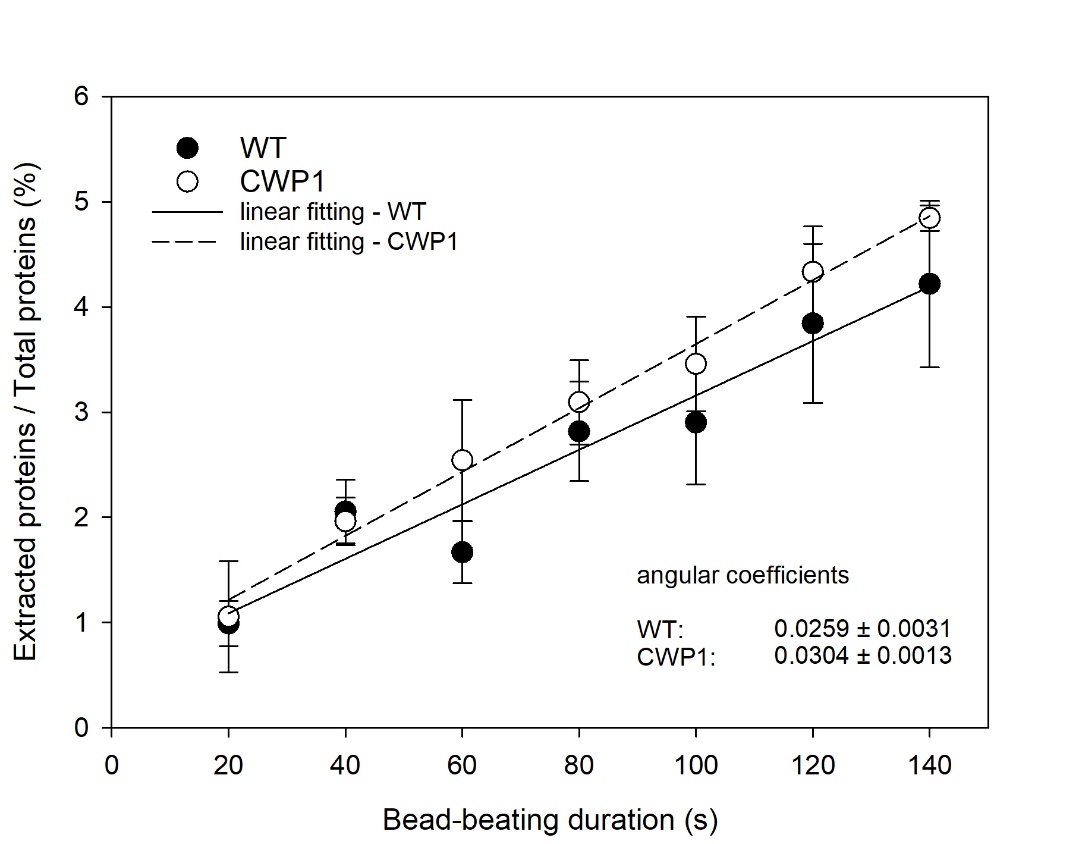
**

**Figure S9. Setup of a bead-beating method for protein extraction from *Cv* biomass.** Aliquots of wet biomass (1·10^8^ *Cv* WT and CWP1 cells) were disrupted by bead-beating with glass beads at 5,000 rpm for 1 to 7 cycles. Each cycle consisted of 20 sec of treatment followed by a 30 sec recovery period. The figure shows the amount of protein found in the supernatant after each cycle, expressed relative to the total protein content in the biomass. Total proteins were extracted from biomass treated with an enzymatic mixture (E1, refer to Figure S2) after 6 cycles of bead-beating and subsequent extraction with a lysis buffer (6% SDS, 6 M Urea, 187 mM Tris-HCl pH 6.8). Protein levels were quantified using the Bradford assay or BCA assay. Data are expressed as mean ± SD, n = 3.

**Figure S10**


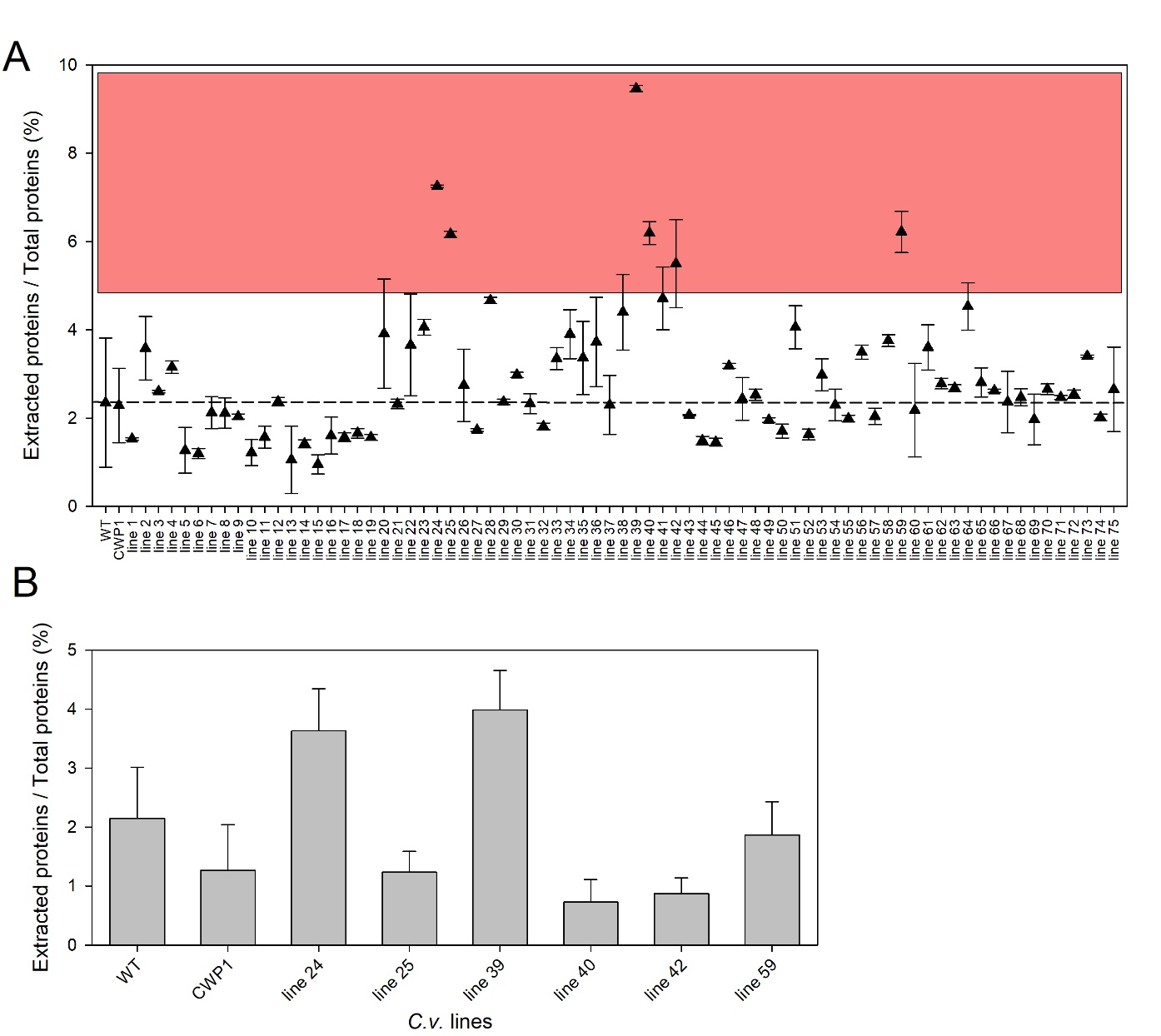


**Figure S10. Characterization of mechanical resistance in *Cv* lines selected for lower Cf binding affinity.** (A) Following flow cytometry analysis (see Figure S8), 75 single colonies were cultured in liquid minimal TAP medium, under illumination at ~80 μmol photons m^-2^ s^-1^ and 24°C for seven days. Samples of 1·10^8^ cells were disrupted via bead-beating at 5,000 rpm (refer to Figure S9), with a total beating time of 60 sec across 3 cycles. Controls included WT and CWP1 cells. Strains exhibiting protein extractability at least twice that of the control genotypes (indicated by the dotted black line) were selected, resulting in 6 lines (highlighted with a pink background), which were further checked for mechanical resistance (B) as in panel A. Data are expressed as mean ± SD, n = 3.

**Figure S11**

**
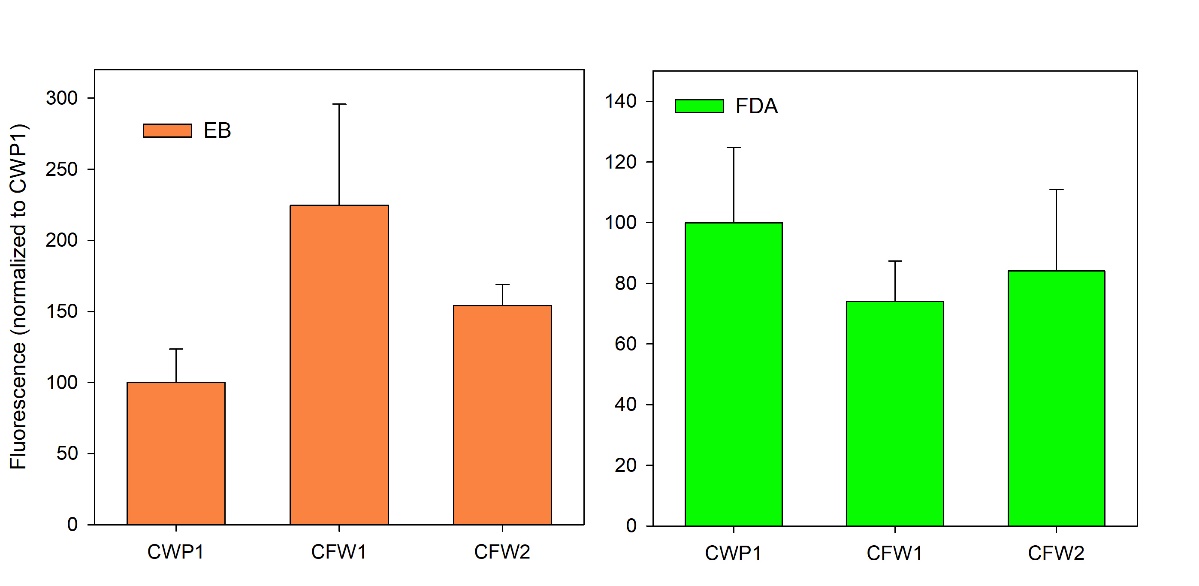
**

**Figure S11. Permeability of CWP1 and CFW cells to fluorescent probes.** *Cv* WT and mutant cells (5·10^6^ cells) were stained with two different fluorescent probes: (A) fluorescein diacetate (FDA) and (B) erythrosine B (EB), to evaluate cell wall permeability. Fluorescence was measured using a plate reader, and the results were normalized to the values obtained from CWP1 cells. Data are expressed as mean ± SD, n = 3.

**Table S1. Data output from sorting performed by the FACSAria Fusion, for isolating CWP mutants.** The sorted samples consisted of *Cv* cells mutagenized with EMS at a concentration ranging from 0 to 3.4%. The following table presents the fluorescent dyes used, the total number of sorted cells, the number of sorted events, and the percentage of events that were FDA+ EB+, FDA+, EB+, and FDA- EB-. Additionally, the number of colony-forming units (CFU) obtained is included.
